# Granulocytic myeloid–derived suppressor cells sustain HIV reservoirs by inhibiting viral reactivation via arginase 1–mediated mechanisms

**DOI:** 10.1172/jci.insight.202628

**Published:** 2026-07-21

**Authors:** Ana Gallego-Cortés, Judith Grau-Expósito, Irene Mota-Gómez, Aleix Benitez-Martinez, Josep Castellvi, Jordi Navarro, Adrian Curran, Joaquin Burgos, Paula Suanzes, Vicenç Falcó, Meritxell Genescà, Maria J. Buzon

**Affiliations:** 1 Infectious Diseases Department, Hospital Universitari Vall d’Hebron (HUVH), Vall d’Hebron Institut de Recerca (VHIR), Universitat Autònoma de Barcelona, Barcelona, Spain.; 2 Department of Pathology, HUVH, Barcelona, Spain.

**Keywords:** AIDS/HIV, Immunology, Monocytes, T cells

## Abstract

Myeloid-derived suppressor cells (MDSCs) represent a heterogeneous population of immature myeloid cells with potent immunosuppressive capabilities that contribute to viral persistence in chronic infections. However, their direct effect on the latent HIV reservoir remains poorly understood. Here, we report that people with HIV (PWH) exhibit elevated levels of MDSCs with notable immunosuppressive activity. Both granulocytic (G-MDSCs) and monocytic (M-MDSCs) subsets expressing arginase 1 (ARG1) or indoleamine 2,3-dioxygenase (IDO) are increased during treated infection, with low-level viral transcription preferentially associated with the expansion of highly suppressive G-MDSCs. Functional assays revealed that G-MDSCs robustly inhibit HIV reactivation from latent reservoirs. Mechanistically, G-MDSCs mediate this inhibition through a contact-independent mechanism, primarily involving ARG1 activity. Our findings demonstrate the capacity of G-MDSCs to sustain HIV reservoirs, suggesting that targeting these cells could potentiate therapeutic strategies aimed at eliminating HIV reservoirs through viral reactivation.

## Abstract 2

G-MDSCs may help HIV persist during treatment by blocking viral reactivation, but targeting ARG1 could improve strategies to reduce the hidden reservoir

## Introduction

Antiretroviral therapy (ART) against the human immunodeficiency virus (HIV) effectively suppresses viral replication in people with HIV (PWH) (1). Adherence to the treatment ensures long-lasting control over the virus, yet it does not lead to its complete elimination (2). The persistence of long-lived cells that are latently infected in the early stage of infection remains the primary obstacle to an HIV cure (3–5).

Spontaneous reactivation of latently infected cells can occur despite viral suppression by ART (6). Multiple factors, such as fluctuations in the host immune response, antigen-driven activation due to vaccination or opportunistic infections, or stochastic events within individual cells, can trigger the activation of these dormant cells and induce viral production (7–10). However, effector immune cells often prove ineffective in targeting reactivated cells (11). HIV highjacks the host immune system, promoting resistance of infected cells to cytotoxic T lymphocyte (CTL) and NK cell–mediated killing (12–14). This compromised immune response is consistent with the lack of latent reservoir decay despite decades of ART (15–17).

Furthermore, HIV-induced immune modulation might affect the efficacy of the “shock and kill” strategy (18). This therapeutic intervention utilizes latency reversal agents (LRAs) to reactivate HIV, ultimately promoting cytopathogenic effects and immune-mediated clearance of infected cells (1, 19, 20). Although several LRAs have successfully induced HIV gene expression from latency in clinical trials (21–25) and ex vivo studies (26–28), they have failed to reduce the latent reservoir size in vivo (21–25, 29, 30). Consequently, identifying the immune-regulatory mechanisms that hinder latency reversal and subsequent effector cell clearance is crucial. Among these regulatory players, myeloid-derived suppressor cells (MDSCs) emerge as compelling candidates.

MDSCs constitute a heterogenous population of immature myeloid cells with potent immunosuppressive capabilities (31), playing a key role in immune regulation across diverse pathological conditions, including cancer, inflammatory diseases, chronic infections, and autoimmune disorders (32). In these settings, MDSC development is driven by the sustained exposure to cytokines and chemokines such as GM-CSF, VEGF, IL-6, IL-1β, TNFα, and CSF1 (32). MDSCs modulate immune responses primarily by suppressing T-lymphocyte activity (33, 34) through several mechanisms, including the expression of arginase 1 (ARG1) and indoleamine 2,3-dioxygenase (IDO) enzymes; the production of reactive nitrogen and oxygen species (NOS and ROS), TGF-β, and IL-10; the upregulation of the immune checkpoint PD-L1; and the induction of Treg expansion (35–39). Phenotypically, MDSCs are identified by the expression of common myeloid markers CD33 and CD11b, alongside the absence or low expression of HLA-DR (31, 40). They are further classified into monocytic-MDSCs (M-MDSCs: CD33^+^ CD11b^+^ HLA-DR^lo/–^ CD14^+^) and granulocytic-MDSCs (G-MDSCs: CD33^dim^ CD11b^+^ HLA-DR^lo/–^ CD14^–^ CD15^+^) according to their lineage of origin (41, 42). However, these markers are not exclusive to MDSCs and are shared with other myeloid cells (43), so functional demonstration of their immunosuppressive capacities is required for definitive identification (42, 43).

Several studies have described the role of MDSCs in HIV infection (44). Elevated frequencies of both M-MDSCs and G-MDSCs have been consistently reported in the bloodstream of PWH during acute and chronic phases of the disease (39, 45–53). This increase in MDSCs correlates with compromised T cell responses (39, 45–51), Treg expansion (39, 45), higher viral loads (VLs) and CD4^+^ T cell depletion (45, 46), reflecting a complex immunomodulatory environment that promotes disease progression and persistence. However, findings regarding MDSC levels during treated infection remain conflicting: some studies report that ART reduces MDSC populations (45, 46, 54), while others observe a sustained elevation despite prolonged treatment (39, 52, 53). Additionally, although ART suppresses plasma viremia, HIV persists in tissues (55–58), potentially promoting MDSC compartmentalization and limiting the accuracy of interpretations based solely on peripheral blood data (59). Therefore, it is critical to elucidate the role of MDSCs in ART-treated individuals, considering their effect in tissues.

MDSCs may contribute to HIV persistence during treatment by maintaining reservoir cells, yet their precise immunosuppressive mechanisms remain uncertain. ARG1 and IDO expression in MDSCs has been implicated in the persistence of tumor cells (37, 38, 60). These enzymes deplete L-arginine and tryptophan, respectively, resulting in the downregulation of the CD3ζ chain expression on activated T cells, leading to T cell hyporesponsiveness and, ultimately, a compromised immune response (61–63). ARG1 activity has been correlated with HIV disease severity (64, 65), and MDSCs from PWH have been found to exhibit elevated ARG1 expression (46). Furthermore, while direct studies on IDO-expressing MDSCs in HIV infection are lacking, heightened IDO activity has been reported in PWH, correlating with disease severity and progression (66–68). Nevertheless, the specific functions of ARG1- or IDO-expressing MDSCs in PWH on ART remain elusive.

In this study, we characterized MDSC subsets and their immunosuppressive properties in PWH. Our findings reveal that HIV infection induces a significant expansion of functional MDSCs, concomitant with augmented immune activation and Treg proliferation. Despite ART decreasing hyperactivation, MDSCs with elevated immunosuppressive capacities persist and are associated with low-level viral transcription. Notably, our results show that G-MDSCs robustly inhibited viral reactivation via an ARG1-mediated mechanism, suggesting a role in sustaining HIV reservoirs during ART. Integrating LRAs with therapeutic approaches targeting MDSC-mediated immunosuppression could represent a promising strategy to disrupt HIV latency and eliminate HIV reservoirs.

## Results

### Differential regulation of MDSC during treated and untreated HIV infection.

**Figure 1:**
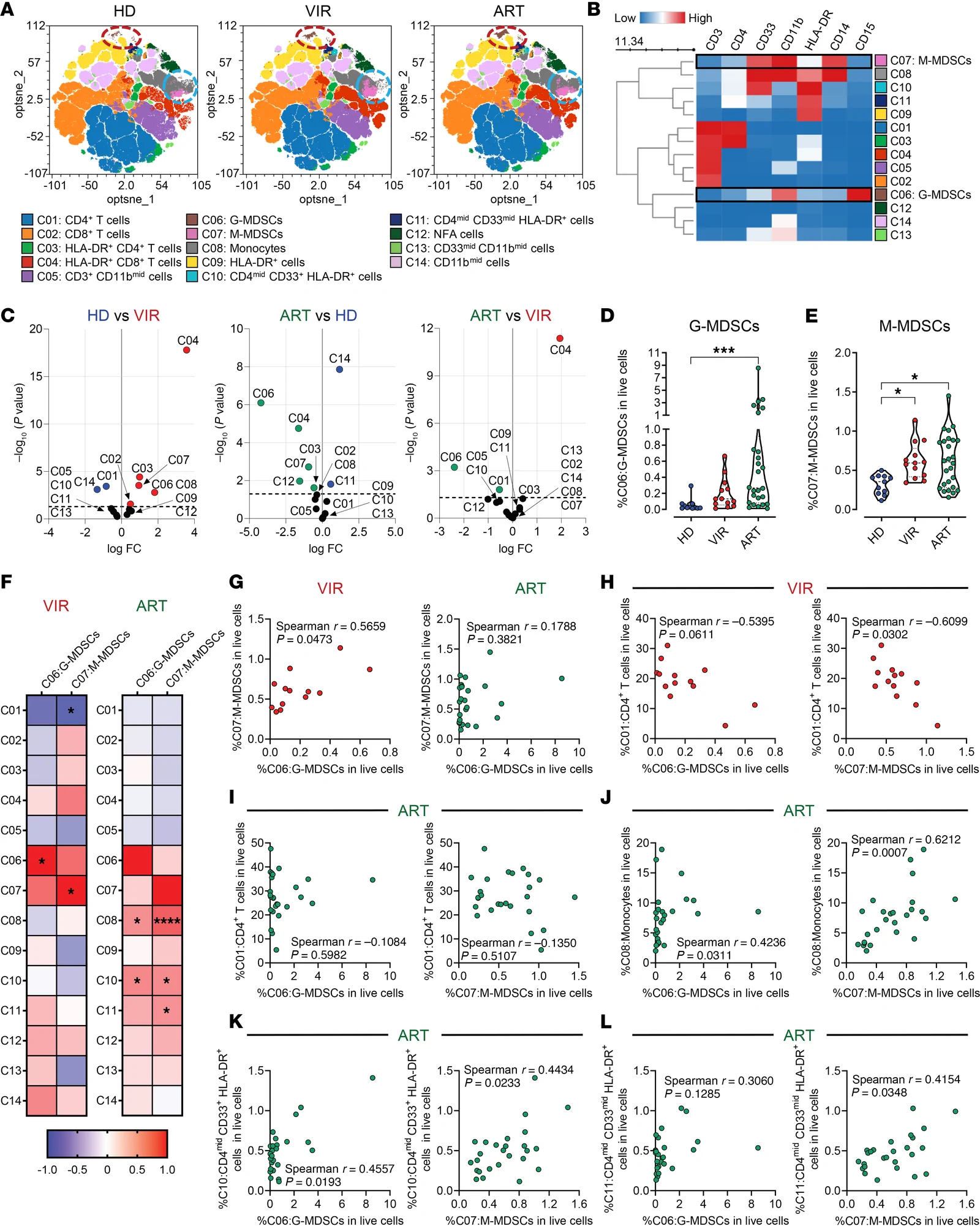
Phenotypic characterization of MDSCs in PWH and HD. (**A**) Opt-SNE plots displaying the distribution of the 14 clusters identified within live PBMCs from HD, VIR, and ART cohorts. G-MDSCs (C06) and M-MDSCs (C07) are highlighted. (**B**) Heatmap illustrating the Mean Fluorescence Intensity (MFI) of CD3, CD4, CD33, CD11b, HLA-DR, CD14, and CD15 markers across clusters. MDSCs were defined as CD3^–^ CD11b^+^ CD33^+^ HLA-DR^–/lo^ cells. G-MDSCs and M-MDSCs were distinguished based on CD33, CD14, and CD15 expression. (**C**) Volcano plots showing significant differences in cluster frequencies between HD, VIR, and ART cohorts. (**D** and **E**) Violin plots displaying the frequency of G-MDSCs (**D**) and M-MDSCs (**E**) within total live PBMCs across groups. Median values with quartiles are represented and statistical comparisons were performed using a 2-sided Kruskal-Wallis test with Dunn’s post hoc correction. (**F**–**L**) Spearman correlation analyses displaying relationships between G-MDSC and M-MDSC subsets and the remaining identified clusters (**F**); each other in the VIR and ART groups (**G**); CD4^+^ T cells (C01) in VIR (**H**) and ART (**I**) individuals; and Monocytes (C08) (**J**), CD4^mid^CD33^+^HLA-DR^+^ cells (C10) (**K**), and CD4^mid^CD33^mid^HLA-DR^+^ cells (C11) (**L**) in the ART cohort. All panels include data from healthy donors (HD, *n* = 11), viremic PWH (VIR, *n* = 13), and ART-suppressed PWH (ART, *n* = 26). *P < 0.05, ***P < 0.001, and ****P < 0.0001. Source data are provided as a Source Data file.

We first compared the frequency of G-MDSCs and M-MDSCs within the peripheral blood of 11 healthy donors (HD), 13 viremic PWH (VIR), and 26 PWH receiving ART with undetectable viremia (ART) (participants HD#1–11, VIR#1–13, and ART#1–26, Supplemental Table 1; supplemental material available online with this article; https://doi.org/10.1172/jci.insight.202628DS1). Flow cytometry was conducted with a panel of markers comprising CD3, CD4, CD33, CD11b, HLA-DR, CD14, and CD15. Unsupervised clustering analysis (FlowSOM) identified 14 distinct clusters shared across all cohorts (Figure 1A) based on the expression patterns of the aforementioned markers (Figure 1B and Supplemental Figure 1). Clusters C06 and C07 represented G-MDSCs (CD3^–^CD4^–^CD33^mid^CD11b^+^HLA-DR^lo^CD14^–^CD15^+^) and M-MDSCs (CD3^–^CD4^–^CD33^hi^CD11b^+^HLA-DR^lo^CD14^+^CD15^–^), respectively. Clusters C01 and C03 were attributed to resting (HLA-DR^–^) and activated (HLA-DR^+^) CD4^+^ T cell populations (CD3^+^CD4^+^), while clusters C02 and C04 delineated the corresponding CD8^+^ T cell subsets (CD3^+^CD4^–^). Cluster C08 was designated as monocytes (CD33^hi^CD11b^+^HLA-DR^+^CD14^+^), and 3 supplementary subsets of CD33^+^ myeloid cells were discerned: C10 (CD4^mid^CD33^+^HLA-DR^+^), C11 (CD4^mid^CD33^mid^HLA-DR^+^), and C13 (CD33^mid^CD11b^mid^). Nonetheless, the remaining 3 clusters, namely C05 (CD3^+^CD11b^mid^), C12 (negative for all [NFA]) and C14 (CD11b^mid^), could not be conclusively characterized due to a lack of lineage-specific markers. As expected, significant differences were observed in the proportions of resting and activated lymphocytes between cohorts (Figure 1C and Supplemental Figure 2). Importantly, elevated frequencies of both MDSC subpopulations were evident in the VIR and ART cohorts relative to the HD group (Figure 1C). Specifically, G-MDSCs (C06) exhibited an increase exceeding 5-fold in the VIR cohort and above 10-fold in the ART group (median_VIR_ = 0.13% and median_ART_ = 0.27%) relative to the HD group (median_HD_ = 0.02%) (Figure 1D). When comparing the 3 cohorts, statistical significance was only achieved between the ART and HD groups (Figure 1D). Regarding M-MDSCs (C07), PWH displayed nearly doubled proportions compared with HD. The highest frequency was observed in the ART group (median_ART_ = 0.61%), followed by the VIR cohort (median_VIR_ = 0.59%), with the HD group exhibiting the lowest frequency (median_HD_ = 0.35%) (Figure 1E). Interestingly, while a significant positive correlation was observed between both MDSC subsets in the VIR group (r_VIR_ = 0.5659, *P*_VIR_ = 0.0473), no such correlation was found in the ART cohort (r_ART_ = 0.1788, *P*_ART_ = 0.3821), suggesting different mechanisms driving G-MDSC and M-MDSC expansion during treatment (Figure 1, F and G). Finally, we determined whether an increase in MDSCs in PWH was associated to changes in the proportion of the other identified clusters (Figure 1F). Notably, both MDSC subsets negatively correlated with resting CD4^+^ T cells (C01) in the VIR group (r_G-MDSCs_ = –0.5385, *P*_G-MDSCs_ = 0.0611; r_M-MDSCs_ = –0.6099, *P*_M-MDSCs_ = 0.0302) (Figure 1H), although such correlations were not observed in the ART group (r_G-MDSCs_ = –0.1084, *P*_G-MDSCs_ = 0.5982; r_M-MDSCs_= –0.1355, *P*_M-MDSCs_ = 0.5107) (Figure 1I). Conversely, in the ART cohort, G-MDSCs and/or M-MDSCs positively correlated with other myeloid cells, including monocytes (C08) (Figure 1J), as well as clusters C10 (Figure 1K) and C11 (Figure 1L).

Overall, we found that productive HIV infection is directly associated with an expansion of MDSCs that persists throughout treated infection. This increase correlates with the CD4^+^ T cell decline characteristic of untreated HIV infection; whereas it coincides with a rise in the frequencies of other myeloid populations during treated infection, yet not with CD4^+^ T cells. This underscores a complex, treatment-dependent interplay between MDSCs and various immune cell populations during HIV infection.

### MDSCs are present in lymph nodes from PWH and are associated with an immunosuppressive microenvironment.

Next, we investigated whether MDSCs could infiltrate and reside within immune-privileged sites and perpetuate HIV persistence. Among these sites, lymph nodes (LNs) serve as crucial HIV sanctuaries and reservoirs due to a distinctive cellular compartmentalization that facilitates the establishment of a persistent infection (69, 70). To discern the role of MDSCs within these lymphoid compartments, we examined the presence and localization of CD33^+^ cells, a validated marker for identifying MDSCs in tissues (71–74), alongside the HIV protein p24 via fluorescence immunostaining in anatomically intact LN sections from VIR and ART individuals (participants VIR#14–16 and ART#50; Supplemental Table 1).

**Figure 2:**
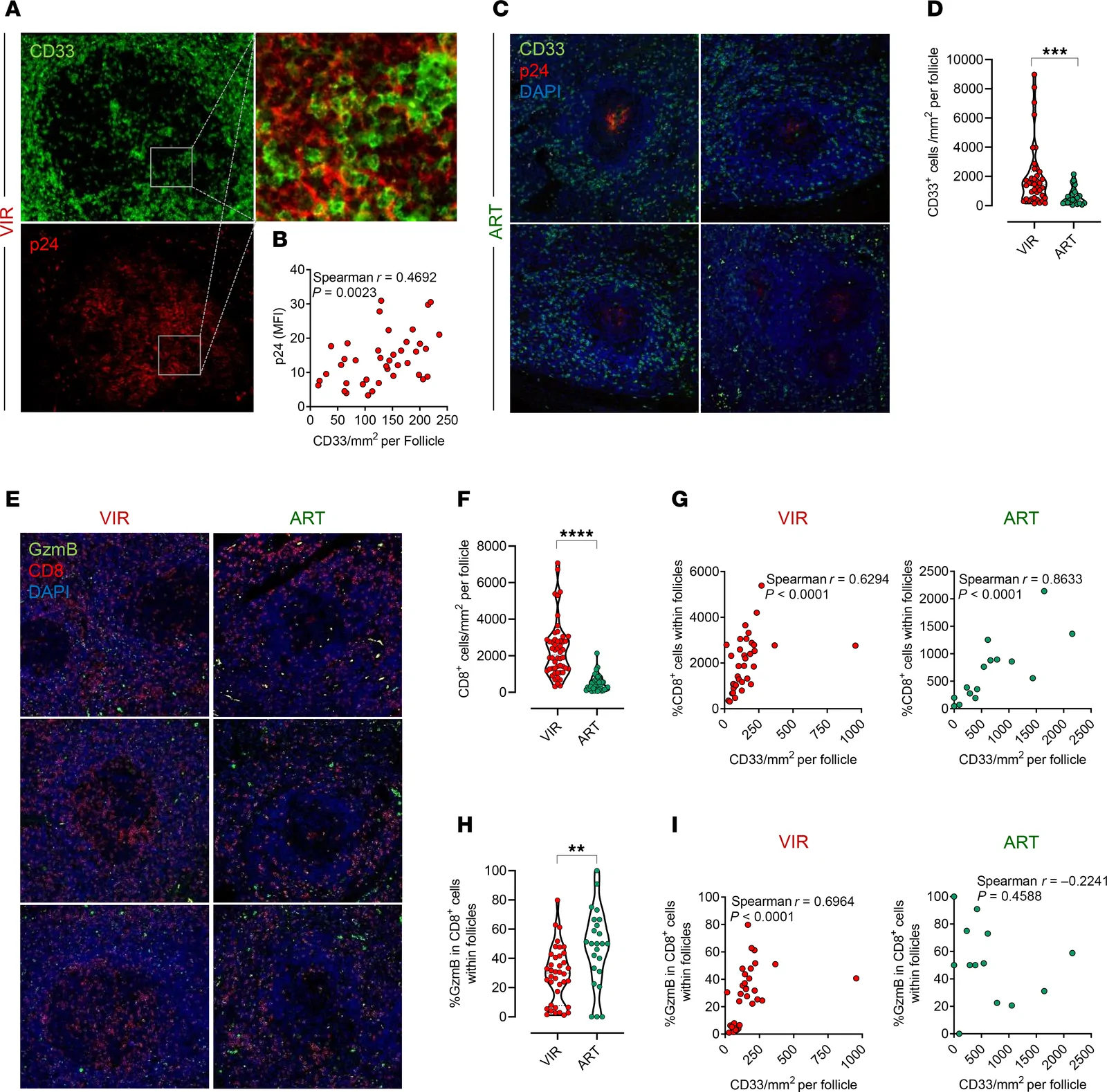
Localization of HIV-infected cells and MDSCs within lymph nodes of VIR and ART individuals. (**A**) Representative micrograph of a lymph node section from a VIR individual stained for CD33 (green) and p24 (red) (×40 magnification). White boxes indicate a region where infected cells (p24^+^) and MDSCs (CD33^+^) colocalize. The right panel shows a zoomed view of this region. (**B**) Correlation between p24 expression, represented as MFI, and CD33^+^ cell density per follicle in lymph node samples from VIR individuals (*n* = 3). (**C**) Representative lymph node sections from an ART individual stained for CD33 (green), p24 (red), and DAPI (blue) (×20 magnification). (**D**) Violin plot showing the density of MDSCs (CD33^+^ cells) within B cell follicles. (**E**) Representative lymph node sections from one VIR and one ART individuals stained for CD8 (red), GzmB (green), and DAPI (blue). (**F**) Violin plot displaying CD8^+^ cell infiltration within B cell follicles. (**G**) Correlations between CD8^+^ and CD33^+^ cells within B cell follicles. (**H**) Violin plot showing the percentage of GzmB expression within CD8^+^ cells in B follicles. (**I**) Correlations between GzmB expression within CD8^+^ cells and CD33^+^ cells in B cell follicles. Unless otherwise indicated, analyses include lymph node samples from VIR (*n* = 3) and ART (*n* = 1) PWH. Statistical comparisons in **D**, **F**, and **H** were performed using 2-sided Mann-Whitney *U* test. **P < 0.01, ***P < 0.001, and ****P < 0.0001. Median values with quartiles are represented in these graphs. Correlations were evaluated by Spearman analysis. Source data are provided as a Source Data file.

In VIR samples, p24 displayed the typical network-like staining pattern, likely representing virions captured by follicular dendritic cells within B cell follicles (Figure 2A). While CD33^+^ cells were predominantly localized in the interfollicular T cell area, their presence within B cell follicles positively correlated with the mean fluorescence intensity (MFI) of p24 (Figure 2B). Conversely, in LNs from ART PWH, despite detecting both p24 and CD33 signals, CD33^+^ cells rarely penetrated the B cell follicle (Figure 2C), demonstrating considerably greater follicular infiltration of MDSCs during uncontrolled compared with treated infection (Figure 2D). IHC staining for CD33 and HLA-DR on LN sections from 1 VIR and 1 ART PWH (participants VIR#17 and ART#50; Supplemental Table 1) confirmed the absence of HLA-DR expression in the CD33^+^ cells, supporting their identity as MDSCs (Supplemental Figure 3).

Subsequently, we examined whether MDSCs within the B cell follicles might foster an immunosuppressive environment that impairs effector T cell function by analyzing the follicular infiltration of CD8^+^ cells and their cytotoxic activity based on granzyme B (GzmB) expression (Figure 2E). For this analysis, we used LN samples from 3 VIR and 1 ART PWH (participants VIR#14–16 and ART#50; Supplemental Table 1). We observed that viremia promoted CD8^+^ cell infiltration into B cell follicles, which was reduced during ART (Figure 2, E and F). Additionally, the follicular presence of CD8^+^ cells strongly correlated with CD33^+^ cells in both VIR and ART (Figure 2G). Notably, GzmB expression in CD8^+^ cells within the B cell follicles was diminished in VIR compared with ART individuals (Figure 2H). Interestingly, GzmB and CD33^+^ staining positively correlated in VIR but not in ART samples (Figure 2I). Taken together, while viremia is likely the primary factor driving the follicular infiltration of MDSCs and CD8^+^ cells, the lower expression of GzmB in CD8^+^ during uncontrolled infection might be due to the increased presence of MDSCs within the B cell follicles.

### MDSCs with enhanced immunosuppressive potential are increased in PWH and persist during ART.

**Figure 3:**
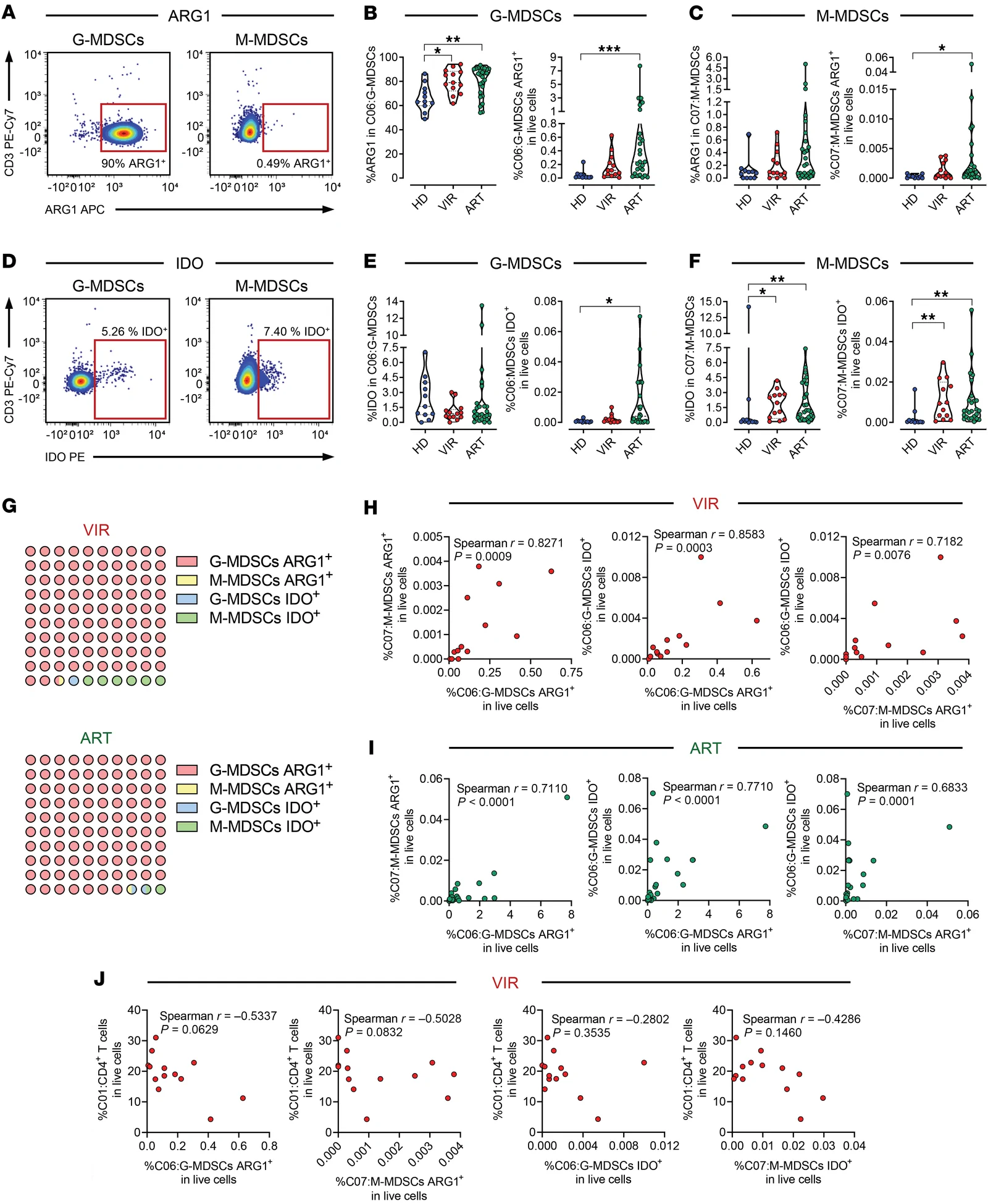
Elevated frequencies of MDSCs with enhanced immunosuppressive capacities in PWH. (**A**) Representative flow cytometry gating examples displaying ARG1 expression in the 2 MDSC subsets studied. (**B**) Violin plots depicting ARG1 expression in G-MDSCs and the proportion of G-MDSCs ARG1^+^ within total live PBMCs from HD, VIR, and ART individuals. (**C**) Violin plots displaying ARG1 expression in M-MDSCs and the proportion of M-MDSCs ARG1^+^ within total live PBMCs across cohorts. (**D**) Representative flow cytometry gating examples illustrating IDO expression in G-MDSCs and M-MDSCs. (**E**) Violin plots showing IDO expression in G-MDSCs and the proportion of G-MDSCs IDO^+^ within total live PBMCs from HD, VIR, and ART individuals. (**F**) Violin plots displaying IDO expression in M-MDSCs and the proportion of M-MDSCs IDO^+^ within total live PBMCs across cohorts. (**G**) Dot plots representing the proportion of ARG1^+^ and IDO^+^ subsets of G-MDSCs and M-MDSCs among total MDSCs expressing ARG1 and/or IDO in the VIR and ART groups. (**H** and **I**) Correlations between G-MDSCs ARG1^+^, M-MDSCs ARG1^+^, and G-MDSCs IDO^+^ in the VIR (**H**) and ART (**I**) cohorts. (**J**) Correlations between CD4^+^ T cells and the MDSC subsets expressing ARG1 or IDO in the VIR group. All panels include data from HD (*n* = 11), VIR (*n* = 13), and ART (*n* = 26) PWH. Statistical comparisons in **B**, **C**, **E**, and **F** were performed using 2-sided Kruskal-Wallis tests with Dunn’s post hoc correction. *P < 0.05, **P < 0.01, and ***P < 0.001. Median values with quartiles are represented in these graphs. Correlations were assessed using Spearman analysis. Source data are provided as a Source Data file.

Next, we evaluated the immunosuppressive potential of MDSCs present in the peripheral blood of HD and PWH (participants HD#1–11, VIR#1–13, and ART#1–26; Supplemental Table 1) by assessing the expression of the immunomodulatory mediators: ARG1 and IDO. In general, ARG1 activity was elevated in G-MDSCs across all cohorts; however, ARG1 expression was significantly upregulated in G-MDSCs from both VIR and ART (median_VIR_ = 79% and median_ART_ = 85.8%), compared with HD (median_HD_ = 63.3%) (Figure 3, A and B). Furthermore, we observed a higher proportion of G-MDSCs ARG1^+^ within total live cells in PWH (median_HD_ = 0.01%; median_VIR_ = 0.11% and median_ART_ = 0.24%), though this was statistically significant only in the ART cohort (Figure 3B). In contrast, ARG1 levels remained low in M-MDSCs across all cohorts (median_HD_ = 0.08%, median_VIR_ = 0.08% and median_ART_ = 0.21%), with only a nonsignificant trend toward upregulation in PWH (Figure 3C). Nonetheless, the frequency of M-MDSCs ARG1^+^ within total live cells was significantly higher in the ART cohort (median_ART_ = 8.8 × 10^–4^%), but not the VIR cohort (median_VIR_ = 5.0 × 10^–4^%) compared with the HD group (median_HD_ = 2.6 × 10^–4^%) (Figure 3C).

Regarding IDO, expression levels were comparable between G-MDSCs and M-MDSCs. G-MDSCs from PWH did not express more IDO than those from HD (median_HD_ = 1.60%; median_VIR_ = 0.87% and median_ART_ = 0.82%); nevertheless, a significant rise in G-MDSCs IDO^+^ within live cells was observed in the ART cohort, but not in the VIR group, compared with the HD group (median_HD_ = 5.5 × 10^–4^%, median_VIR_ = 1.1 × 10^–3^%, and median_ART_ = 4.1 × 10^–3^%) (Figure 3, D and E). Conversely, M-MDSCs from both VIR and ART individuals exhibited significantly higher IDO expression than those from the HD cohort (median_HD_ = 0.10%, median_VIR_ = 1.97% and median_ART_ = 1.16%) (Figure 3F). Furthermore, the percentage of M-MDSCs IDO^+^ was elevated in both VIR and ART groups when compared with the HD cohort (median_HD_ = 3.9 × 10^–4^%, median_VIR_ = 9.4 × 10^–3^%, and median_ART_ = 5.7 × 10^–3^%) (Figure 3F).

Among the immunosuppressive MDSC subsets studied, G-MDSCs ARG1^+^ were the most prevalent subpopulation in both VIR and ART cohorts (median_VIR_ = 92.5% and median_ART_ = 95%), followed by M-MDSCs IDO^+^ (median_VIR_ = 6% and median_ART_ = 1.5%), while G-MDSCs IDO^+^ (median_VIR_ = 1% and median_ART_ = 1%) and M-MDSCs ARG1^+^ (median_VIR_ = 0.5% and median_ART_ = 0.5%) were present at much lower frequencies (Figure 3G). This distribution was similar in HD individuals (Supplemental Figure 4A). Moreover, the frequencies of G-MDSCs ARG1^+^, M-MDSCs ARG1^+^, and G-MDSCs IDO^+^ strongly and positively correlated with one another in both VIR and ART cohorts (Figure 3, H and I, and Supplemental Figure 4B). In contrast, M-MDSCs IDO^+^ did not correlate with any other subset (Supplemental Figure 4B). Additionally, a negative trend was observed between both G-MDSCs ARG1^+^ and M-MDSCs ARG1^+^ frequencies and the percentage of CD4^+^ T cells in the VIR group, whereas this trend was less pronounced for IDO-expressing subsets (Figure 3J).

Overall, we found distinct expression of ARG1 and IDO in G-MDSCs and M-MDSCs in response to HIV infection. Heightened expression of ARG1 was detected exclusively in G-MDSCs, while IDO was solely increased in M-MDSCs from PWH. Moreover, all these immunosuppressive subsets were more abundant in PWH on ART than in HD individuals.

### Chronic immune activation under ART is associated with increased Treg levels but not with MDSC persistence.

**Figure 4:**
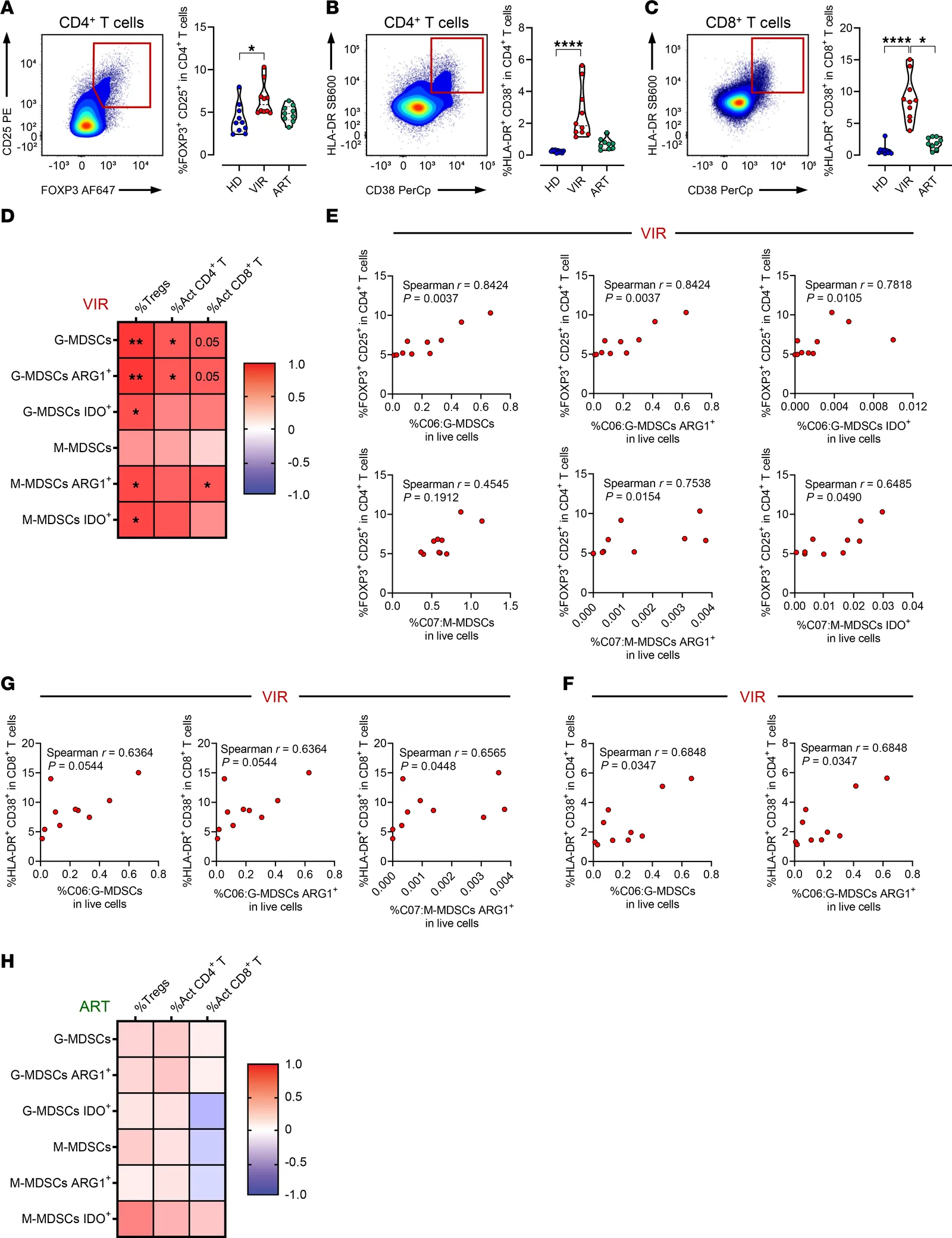
Expansion and persistence of highly immunosuppressive MDSCs in the dysregulated immune environment of chronic HIV infection. (**A**) Violin plots displaying the frequency of FOXP3^+^CD25^+^CD4^+^ T cells (Tregs) within total CD4^+^ T cells across HD, VIR, and ART cohorts. (**B** and **C**) Violin plots showing HLA-DR and CD38 expression in CD4^+^ T cells (**B**) and CD8^+^ T cells (**C**) from HD, VIR, and ART individuals. (**D**) Correlations between Tregs, as well as chronic activation in both CD4^+^ T cells and CD8^+^ T cells, and the G-MDSCs (C06) and M-MDSCs (C07) subsets in the VIR cohort. (**E**) Correlations between Tregs within total CD4^+^ T cells and all subsets of MDSCs in VIR individuals. (**F**) Correlations between G-MDSCs and G-MDSCs ARG1^+^, and activation in CD4^+^ T cells in the VIR group. (**G**) Correlations between G-MDSCs, G-MDSCs ARG1^+^ and M-MDSCs ARG1^+^, and activation in CD8^+^ T cells in the VIR cohort. (**H**) Correlations between Tregs, as well as chronic activation in both CD4^+^ T cells and CD8^+^ T cells, and the G-MDSCs (C06) and M-MDSCs (C07) subsets in the ART cohort. Statistical comparisons in **A**–**C** were performed using the 2-sided Kruskal-Wallis test with Dunn’s post hoc correction. *P < 0.05, **P < 0.01, and ****P < 0.0001. Median values with quartiles are depicted in the graphs. Unless otherwise indicated, analyses include samples from 10 HD, 10 VIR, and 9 ART individuals. Correlations were assessed using Spearman analysis. Source data are provided as a Source Data file.

Chronic HIV infection induces the expansion of immunosuppressive cells, including FOXP3^+^CD25^+^CD4^+^ Tregs and MDSCs (44, 75–77). Despite the presence of these suppressor cells, immune activation markers (HLA-DR, CD38) on CD4^+^ and CD8^+^ T cells remain elevated even under effective ART (78, 79). To assess the association between MDSC subsets and this dysregulated immune environment, we used a flow cytometry panel including markers for T cells (CD3, CD4, CD8), Tregs (CD25, FOXP3), and T cell activation (HLA-DR, CD38) (Supplemental Figure 5). We analyzed samples from 19 PWH (VIR = 10 and ART = 9) and 10 HD individuals (participants HD#12–21, VIR#1, 4–10, 12, and 13, and ART#1, 2, 5–8, 17, 22, and 25; Supplemental Table 1) and identified Tregs and chronically activated CD4^+^ and CD8^+^ T cells based on the aforementioned markers (Supplemental Figure 5, A and B). Consistent with previous reports (75, 76, 78, 79), VIR PWH exhibited a significant increase in Treg frequencies (median_VIR_ = 5.90%; median_HD_ = 3.78%), alongside elevated HLA-DR and CD38 expression in both CD4^+^ T cells (median_HD_ = 0.25%, median_VIR_ = 1.85%) and CD8^+^ T cells (median_HD_ = 0.54%, median_VIR_ = 8.49%) compared with HD individuals (Figure 4, A–C). Treg frequencies and T cell activation levels remained higher in the ART cohort than in the HD group (median_ART-_Treg = 4.86%; median_ART-Act_ _CD4_ = 0.68%; median_ART-Act_ _CD8_ = 1.85%) (Figure 4, A–C). Additionally, we observed positive trends between Treg frequencies and chronic T cell activation, as well as between HLA-DR and CD38 coexpression in CD4^+^ T cells and in CD8^+^ T cells in both PWH cohorts (Supplemental Figure 6).

We subsequently examined whether G-MDSCs and M-MDSCs and their respective ARG1^+^ or IDO^+^ subsets correlated with Treg frequencies and chronic immune activation in PWH (Figure 4D). In the VIR cohort, we observed strong positive trends between all MDSC subsets and Tregs (Figure 4, D and E). Likewise, positive correlations were detected between all MDSC subsets and chronic T cell activation (Figure 4D). Specifically, HLA-DR and CD38 expression in CD4^+^ T cells robustly and significantly correlated with total G-MDSCs (r_G-MDSCs_ = 0.6848, *P*_G-MDSCs_ = 0.0347) and G-MDSCs ARG1^+^ (r_G-MDSCs_ _ARG1+_ = 0.6848, *P*_G-MDSCs_ _ARG1+_ = 0.0347) (Figure 4F). Similarly, HLA-DR^+^ CD38^+^ CD8^+^ T cells were positively associated with total G-MDSCs (r_G-MDSCs_ = 0.6364, *P*_G-MDSCs_ = 0.0544), G-MDSCs ARG1^+^ (r_G-MDSCs_ _ARG1+_ = 0.6364, *P*_G-MDSCs_ _ARG1+_ = 0.0544) and M-MDSCs ARG1^+^ (r_M-MDSCs_ _ARG1+_ = 0.6565, *P*_M-MDSCs_ _ARG1+_ = 0.0448) (Figure 4, D and G). However, only the correlation with M-MDSCs ARG1^+^ reached statistical significance (Figure 4, D and G). These results suggest that chronic immune activation during uncontrolled HIV infection is accompanied by the expansion of Tregs and MDSCs with enhanced immunosuppressive capabilities.

In contrast, in the ART cohort, where T cell activation, Tregs, and MDSC frequencies remained elevated relative to the HD group (Figure 4, A–C, and Figure 1D), MDSCs displayed no robust positive correlations with either Tregs or HLA-DR and CD38 expression in CD4^+^ T and CD8^+^ T cells (Figure 4H). These findings suggest that, while the highly inflammatory environment of untreated viremia coordinates Treg and MDSC expansion, chronic low-level immune activation during ART does not drive MDSC persistence.

### G-MDSCs are linked to HIV persistence during treatment.

To further define the role of MDSCs in HIV pathogenesis, we examined associations between MDSC abundance and markers of viral burden, reservoir size, and clinical parameters in PWH (participants VIR#2–13 and ART#1–3, 5, 7, 8, 13, 17, and 19–26; Supplemental Table 1). Initially, we quantified intracellular HIV RNA and DNA across cohorts, revealing higher levels of both in VIR individuals, as expected (Supplemental Figure 7, A–C). HIV RNA and DNA levels were inversely associated with CD4^+^ T cell frequencies in both cohorts (Supplemental Figure 7D). We next assessed the HIV reservoir in ART PWH by quantifying intact HIV DNA (Supplemental Figure 7E). Given potential differences in MDSC-mediated immunosuppression between active viral replication and treated infection, VIR and ART individuals were analyzed separately.

**Figure 5:**
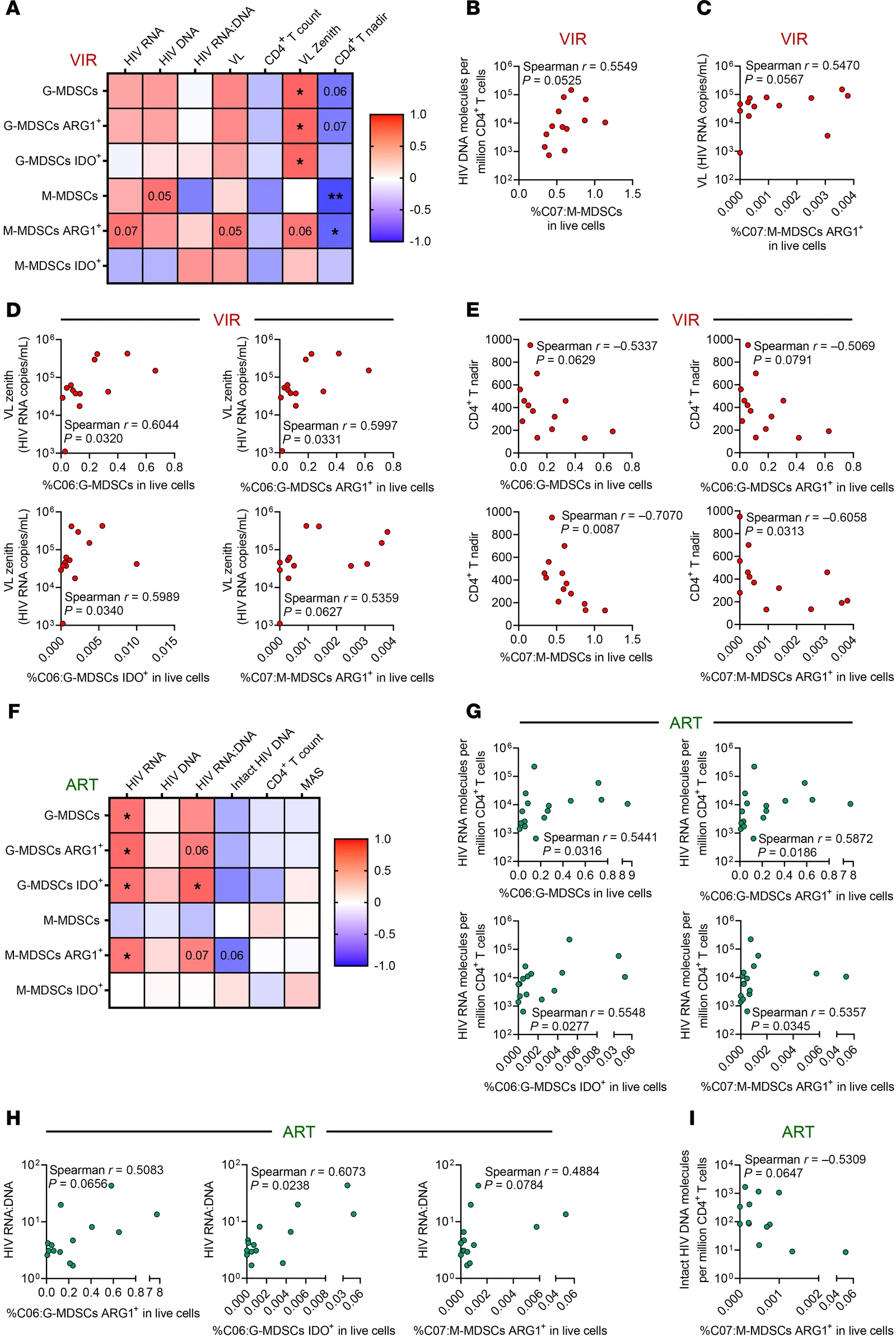
MDSCs expand during acute HIV infection and are maintained during ART associated with low-level viral transcription. Spearman correlation analysis displaying relationships in the VIR cohort between: (**A**) G-MDSCs and M-MDSCs, as well as their ARG1^+^ and IDO^+^ subsets; HIV RNA (*n* = 12) and HIV DNA (*n* = 13) within CD4^+^ T cells; the HIV RNA/DNA ratio (*n* = 12); Viral Load (VL) (*n* = 13); CD4^+^ T cell count (*n* = 13); VL zenith (*n* = 13); and nadir CD4^+^ T cell count (*n* = 13). (**B**) M-MDSCs and HIV DNA in CD4^+^ T cells (*n* = 13). (**C**) M-MDSCs ARG1^+^ and VL (*n* = 13). (**D**) All G-MDSC subsets and M-MDSCs ARG1^+^ and VL zenith (*n* = 13). (**E**) G-MDSCs, M-MDSCs and their ARG1^+^ subsets, and nadir CD4^+^ T cell count (*n* = 13). Spearman correlation analysis displaying relationships in the ART cohort between: (**F**) all MDSC subsets and HIV RNA (*n* = 16) and HIV DNA (*n* = 14) within CD4^+^ T cells, the HIV RNA/DNA ratio (*n* = 14), intact HIV DNA in CD4^+^ T cells (*n* = 13), CD4^+^ T cell count (*n* = 26), and months on suppressive ART (MAS) (*n* = 26). (**G**) G-MDSCs, G-MDSCs ARG1^+^, G-MDSCs IDO^+^ and M-MDSCs ARG1^+^, and HIV RNA within CD4^+^ T cells (*n* = 16). (**H**) G-MDSCs ARG1^+^, G-MDSCs IDO^+^, and M-MDSCs ARG1^+^ and the HIV RNA:DNA ratio (*n* = 14). (**I**) M-MDSCs ARG1^+^ and intact HIV DNA in CD4^+^ T cells (*n* = 13). Source data are provided as a Source Data file.

In the VIR cohort, we found no statistically significant associations between MDSCs ARG1^+^ or IDO^+^ and HIV RNA, HIV DNA, or HIV RNA/DNA ratio (Figure 5A). However, there was a trend toward a positive correlation between total M-MDSCs and HIV DNA (r_M-MDSCs_ = 0.5549, *P*_M-MDSCs_ = 0.0525) (Figure 5B), suggesting an expansion of M-MDSCs during untreated infection that may contribute to viral reservoir establishment and persistence. Moreover, IDO expression in M-MDSCs positively correlated with the HIV RNA:DNA ratio (r_IDO_ _in_ _M-MDSCs_ = 0.6713, *P*_IDO_ _in_ _M-MDSCs_ = 0.0202) (Supplemental Figure 7F), linking upregulated IDO activity to increased HIV transcriptional activity.

Subsequently, we evaluated correlations between MDSCs and key prognostic markers of HIV disease. M-MDSCs ARG1^+^ frequency and ARG1 expression levels within M-MDSCs were positively associated with plasma VL (r_M-MDSCs_ _ARG1+_ = 0.5470, *P*_M-MDSCs_ _ARG1+_ = 0.0567; r_ARG1+_ _in_ _M-MDSCs_ = 0.5249, p_ARG1+_ _in_ _M-MDSCs_ = 0.0690) (Figure 5C and Supplemental Figure 7F), implying that this specific immunoregulatory MDSC subset may exert a direct influence on the progression of HIV infection or arise as a consequence thereof. Notably, G-MDSCs and their ARG1^+^ or IDO^+^ subsets robustly and positively correlated with peak plasma VL or VL zenith (defined as the highest viral load value observed during follow-up) (r_G-MDSCs_ = 0.6044, *P*_G-MDSCs_ = 0.0320; r_G-MDSCs-ARG1+_ = 0.5997, *P*_G-MDSCs-ARG1+_ = 0.0331; r_G-MDSCs-IDO+_ = 0.5989, *P*_G-MDSCs-IDO+_ = 0.0340) (Figure 5, A and D). Trends toward positive associations were also observed between the frequency of M-MDSC ARG1^+^ and ARG1 expression in M-MDSCs and VL zenith (r_M-MDSCs-ARG1+_ = 0.5359, *P*_M-MDSCs-ARG1+_ = 0.0627; r_ARG1+_ _in_ _M-MDSCs_ = 0.5259, *P*_ARG1+_ _in_ _M-MDSCs_ = 0.0627) (Figure 5D and Supplemental Figure 7F). These associations were coupled with negative correlations with nadir CD4^+^ T cell counts for some subsets (r_G-MDSCs_ = –0.5337,*P*p_G-MDSCs_ = 0.0629; r_G-MDSCs-ARG1+_ = –0.5069, *P*_G-MDSCs-ARG1+_ = 0.0791; r_M-MDSCs-ARG1+_ = –0.6058, *P*_M-MDSCs-ARG1+_ = 0.0313) (Figure 5E). Interestingly, although M-MDSCs did not correlate with VL zenith, they showed a strong inverse association with nadir CD4^+^ T cells (r_M-MDSCs_ = –0.7070, *P*_M-MDSCs_ = 0.0087). These results hint at a marked expansion of specific immunosuppressive MDSC subsets during the replicative phase of the infection, linking active viral replication to the generation of these subpopulations.

Next, our focus shifted toward characterizing MDSCs in the context of treated infection. For this analysis, we used samples from ART individuals on suppressive drug regimens for a median duration of 49 months (Supplemental Table 1). All G-MDSC subsets and M-MDSCs ARG1^+^ positively correlated with HIV RNA levels within CD4^+^ T cells (r_G-MDSCs_ = 0.5643, *P*_G-MDSCs_ = 0.0310; r_G-MDSCs-ARG1+_ = 0.6130, *P*_G-MDSCs-ARG1+_ = 0.0171; r_G-MDSCs-IDO+_ = 0.5594, *P*_G-MDSCs-IDO+_ = 0.0324; r_M-MDSCs-ARG1+_ = 0.5576, *P*_M-MDSCs-ARG1+_ = 0.0330) (Figure 5, F and G), suggesting a link between MDSCs, particularly G-MDSCs, and HIV transcriptional activity within the CD4^+^ T cell compartment during ART. Similar trends were observed for the HIV RNA/DNA ratio (Figure 5, F and H), supporting an association between MDSCs and per-cell low-level transcription. Moreover, ARG1 expression in M-MDSCs positively correlated with both HIV RNA (r_ARG1+_ _in_ _M-MDSCs_ = 0.5798, , *P*_ARG1+_ _in_ _M-MDSCs_ = 0.0204) and the HIV RNA:DNA ratio (r_ARG1+_ _in_ _M-MDSCs_ = 0.5501, , *P*_ARG1+_ _in_ _M-MDSCs_ = 0.0442) (Supplemental Figure 7G), indicating that ARG1 activity in this subset may be driven by residual viral transcription. We next examined associations with total and intact HIV DNA. No significant correlations were observed between MDSC subsets and either measure, except for a near-significant inverse trend between M-MDSCs ARG1^+^ and intact HIV DNA (r_M-MDSCs-ARG1+_ = –0.5309, *P*_M-MDSCs-ARG1+_ = 0.0647) (Figure 5, F and I). These findings suggest that MDSC abundance under ART is driven by low-level viral transcription rather than overall reservoir size.

Furthermore, individuals with higher CD4^+^ T cell counts and longer durations on suppressive ART (Months ART-suppressed [MAS]) did not display reduced MDSC frequencies (Figure 5F). Notably, IDO expression in G-MDSCs showed an inverse association with CD4^+^ T cell frequency (r_IDO_ _in_ _G-MDSCs_ = –0.4446, *P*_IDO_ _in_ _G-MDSCs_ = 0.0539) and absolute counts (r_IDO_ _in_ _G-MDSCs_ = –0.4752, *P*_IDO_ _in_ _G-MDSCs_ = 0.0142) (Supplemental Figure 7G), suggesting that reduced IDO activity in this subset may accompany improved immune reconstitution. Overall, these findings indicate that MDSC levels remain elevated despite suppressive ART and may be sustained in part by residual viral transcriptional activity.

### G-MDSCs from PWH inhibit HIV reactivation from the latent reservoir in a contact-independent manner.

**Figure 6:**
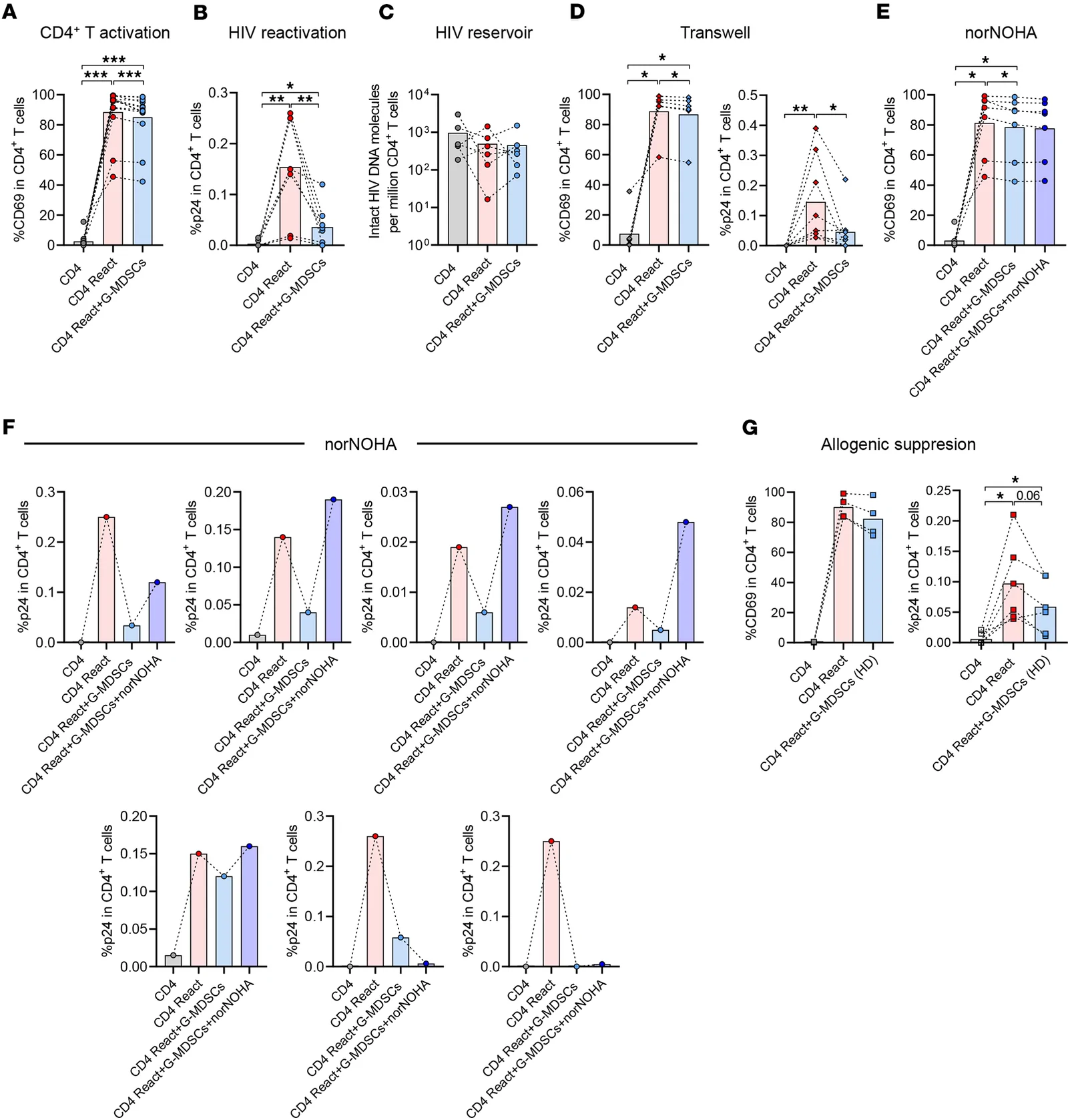
G-MDSCs decrease CD4^+^ T-cell activation and HIV reactivation. Ex vivo reactivated CD4^+^ T cells (CD4 React) from ART PWH were cocultured overnight with G-MDSCs, after which CD4^+^ T cell activation and viral reactivation were measured. Graphs showing: (**A** and **B**) CD69 expression (*n* = 13) (**A**) and intracellular p24 levels (*n* = 8) (**B**) within CD4^+^ T cells after coculture with autologous G-MDSCs. (**C**) Intact HIV DNA levels in CD4^+^ T cells unstimulated and following reactivation in the presence or absence of autologous G-MDSCs (*n* = 6). (**D**) Impact on CD4^+^ T cell activation (*n* = 6) and p24 levels within CD4^+^ T cells (*n* = 8) when CD4^+^ T cells and G-MDSCs were cultured in a transwell system. (**E** and **F**) Effect of the ARG1 inhibitor nor-NOHA on CD4^+^ T cell activation (*n* = 7) (**E**) and intracellular p24 expression (*n* = 7) (**F**) when added to the CD4^+^ T cell-MDSC coculture. (**G**) CD69 (*n* = 4) and p24 expression (*n* = 6) in reactivated CD4^+^ T cells from ART PWH cultured alone or in the presence of G-MDSCs from HD individuals. In all panels, each point represents an individual donor from independent assays and lines connect paired mono- and cocultures. Circles indicate experiments in which autologous CD4^+^ T cells and G-MDSCs were cultured in direct contact, diamonds indicate transwell cultures, and squares represent heterologous cultures using G-MDSCs from HD individuals. All experiments were independently replicated, with each run including PBMC samples from distinct donors. Statistical comparisons were performed using 2-sided Wilcoxon tests with significance levels denoted as *P < 0.05; **P < 0.01; ***P < 0.001; ****P < 0.0001. Mean values are represented in these graphs. Source data are provided as a Source Data file.

MDSCs may perpetuate HIV reservoirs by limiting viral reactivation. Given that G-MDSCs exhibited the strongest association with markers of viral persistence, we conducted functional ex vivo coculture assays using isolated G-MDSCs and autologous, pharmacologically reactivated CD4^+^ T cells from ART PWH (participants ART#10–12, 14, 16–18, 27, and 29–37; Supplemental Table 1). Following a previously validated method (14, 80), we evaluated CD4^+^ T cell activation via surface CD69 expression and viral reactivation by measuring intracellular HIV p24 levels, after PMA and Ionomycin stimulation (Supplemental Figure 8). G-MDSCs elicited a modest yet significant reduction (median decrease: 4.1%) in CD69 expression on CD4^+^ T cells (Figure 6A). Crucially, G-MDSCs significantly decreased intracellular p24 levels (median reduction: 79.1%), indicating that they could impede viral reactivation from the latent reservoir (Figure 6B). To further assess whether G-MDSCs contribute to reservoir maintenance, we quantified intact HIV DNA by IPDA in CD4^+^ T cells left unstimulated or following reactivation in the presence or absence of G-MDSCs (participants ART#19 and 45–49; Supplemental Table 1), observing no statistically significant differences in intact HIV DNA levels between conditions. Together, our data suggest that G-MDSCs limit viral inducibility, potentially contributing to reservoir persistence.

Next, to determine whether G-MDSC–mediated inhibition of viral reactivation was contact dependent, we conducted coculture experiments in transwells (participants ART#11, 16, 18, 28–31, and 36; Supplemental Table 1). G-MDSCs reduced both CD69 expression and intracellular p24 levels in the transwell setup (Figure 6D), demonstrating that their suppressive effect is contact independent. To elucidate the underlying mechanism, we investigated which soluble factor–mediated mechanism could be involved in this inhibition. Given the elevated ARG1 expression in G-MDSCs from ART individuals, we hypothesized that this enzymatic activity could contribute to this suppression. To test this possibility, G-MDSC–T cell coculture experiments were performed in the presence of the ARG1 inhibitor nor-NOHA (37, 81–83). Remarkably, while ARG1 inhibition had no significant effect on CD4^+^ T cell activation (Figure 6E), HIV reactivation was almost completely restored in most assays (Figure 6F), identifying ARG1 activity as a key mediator of G-MDSC–driven suppression of viral reactivation.

Furthermore, to assess whether the observed suppressive activity is specific to G-MDSCs from PWH, we performed allogeneic coculture assays pairing reactivated CD4^+^ T cells from ART PWH with G-MDSCs isolated from HD (participants HD#22–27 and ART#38–44; Supplemental Table 1). HD-derived G-MDSCs also exhibited a trend toward reducing both CD4^+^ T cell activation and HIV reactivation (Figure 6G). These findings are consistent with our previous observations implicating ARG1 activity in viral reactivation suppression, as G-MDSCs from HD express baseline high levels of ARG1 (Figure 3B). Collectively, these results suggest that the suppression of HIV reactivation is driven by an intrinsic enzymatic property of G-MDSCs rather than an HIV-specific mechanism.

Overall, our results indicate that G-MDSCs can reduce CD4^+^ T cell activation and inhibit HIV reactivation in a contact-independent manner, with G-MDSC–derived ARG1 activity serving as a pivotal mediator of this suppression.

## Discussion

Despite the effectiveness of ART, HIV persists within latently infected cells that remain largely invisible to the immune system (84) unless reactivated spontaneously (6) or pharmacologically (20). Latency reversal leads to the production of infectious virions that effector immune cells can target, but HIV induces immunomodulatory mechanisms that impair reactivation kinetics and effector cell functions, thereby hindering viral elimination (12–14, 85). MDSCs have emerged as key regulatory players implicated in disease progression in PWH (39, 45, 52, 53). These immature myeloid cells possess potent immunosuppressive capacities that may contribute to HIV reservoir maintenance. In this study, we characterized the distinct immunosuppressive attributes of the 2 main peripheral MDSC subsets in PWH. Our findings reveal the presence of MDSCs with elevated immunosuppressive capabilities despite viral suppression. Notably, we showed that G-MDSCs substantially inhibit viral reactivation via an ARG1-dependent pathway, suggesting their role in sustaining HIV persistence.

Previous studies have consistently reported elevated MDSC frequencies in the peripheral blood of PWH with productive infection (44), although evidence remains conflicting regarding whether this expansion primarily involves G-MDSCs (45, 47–49, 51–53) or M-MDSCs (39, 46, 50). Our findings not only reveal increased levels of both subpopulations in untreated PWH but also demonstrate a robust correlation between them. In agreement with existing literature (45, 46, 49), MDSC frequencies correlated negatively with CD4^+^ T cell percentages, linking their expansion to disease progression. In ART PWH, reports are likewise inconsistent; some describe that ART effectively diminishes MDSCs (45, 46, 53, 54), while others argue they remain elevated (39, 52, 86). Furthermore, even when a decline was reported, MDSC levels failed to normalize to those observed in HD (46, 53). Our cross-sectional data further challenge the notion that ART reduces MDSC abundance, demonstrating that these cells persist at frequencies comparable to or exceeding those of untreated PWH.

MDSCs are typically characterized by their immunosuppressive properties. Our study specifically examined the immunomodulatory enzymes ARG1 and IDO, both of which have been associated with disease severity (64–67). Although upregulation of ARG1 and IDO in MDSCs has been documented in various pathological conditions (87–93), whether HIV infection selectively enhances their expression to exacerbate immunosuppression remains unclear. We found significantly higher ARG1 activity exclusively in G-MDSCs from PWH, a phenomenon observed in oncological settings (90, 91). Conversely, IDO upregulation was restricted to M-MDSCs among PWH, implicating this subset in the increased IDO activity observed during HIV infection. Importantly, both ARG1- and IDO-expressing G-MDSCs and M-MDSCs were more abundant in PWH, particularly in ART individuals, than in HD, suggesting a potential contribution of these populations to HIV persistence.

Previous studies have linked MDSC expansion during HIV infection directly to active viral replication (45, 46, 49), identifying specific viral proteins driving this proliferation (39, 46, 50). In our cohort, we found no significant correlations between concurrent MDSC frequencies and plasma VL; however, most subsets, particularly G-MDSCs, positively associated with VL zenith and negatively correlated with nadir CD4^+^ T cell counts. Together with previous reports (49), these findings suggest that HIV replication during early infection triggers an initial MDSC expansion that persists into the chronic stage. This expansion is likely fueled by the surge of proinflammatory cytokines characteristic of the acute phase, some of which may sustain MDSC persistence throughout chronic infection (44, 50, 51). In the context of treated infection, most MDSC subsets, especially G-MDSCs, positively correlated with HIV transcriptional activity within the CD4^+^ T cell compartment. We hypothesize that inflammatory mediators induced by residual viremia could be reinforcing the maintenance and expansion of MDSCs during ART (52, 86).

MDSCs may also reside within tissues that serve as key HIV reservoirs, consistent with their well-documented ability to infiltrate tumors and promote immune tolerance (94, 95). Indeed, MDSC expansion has been reported in the genital tract of women with cervical infection (59) and in PWH with anal dysplasia (96). In our study, we identified MDSCs in secondary LNs of PWH and demonstrated their infiltration into B cell follicles during productive infection, a phenomenon diminished by ART. Concurrently, CD8^+^ cells within B cell follicles exhibited reduced cytotoxic activity. Although these findings are consistent with an MDSC-mediated immunosuppressive microenvironment, our immunohistochemical data are based on a limited number of archived samples and should be interpreted as supportive rather than definitive evidence. Nevertheless, it is plausible that the persistence of HIV in Tfh cells (97, 98) is facilitated by a compromised cytotoxic response driven by follicular MDSCs. Furthermore, MDSC infiltration into the B cell area may also be associated with impaired Tfh function and delayed antibody responses, hallmarks of untreated HIV infection (99). Mechanistically, MDSCs might regulate Tfh proliferation and activity via the PD1/PDL1 pathway, given the characteristic upregulation of PD1 on Tfh cells (100).

MDSCs may contribute to HIV persistence by inhibiting effector T cell function, thereby limiting clearance of infected cells (45, 46, 48–50). However, whether MDSCs directly modulate viral latency remained uncharacterized. We found that G-MDSCs inhibited HIV reactivation ex vivo without altering overall reservoir size following pharmacological stimulation, suggesting a potent mechanism promoting CD4^+^ T cell anergy and maintaining viral latency. Since these experiments relied on strong PMA/ionomycin stimulation, further investigation is needed to determine whether this suppression varies in the presence of alternative, clinically relevant LRAs. Mechanistically, this inhibition of reactivation was contact independent, occurring in transwell settings and pointing to the involvement of a soluble factor. Given the high baseline ARG1 expression in G-MDSCs, we hypothesized that local depletion of L-arginine drives this effect (37, 46, 101). Confirming this, treatment of G-MDSCs with nor-NOHA, an ARG1 inhibitor with demonstrated efficacy (37, 81–83, 102), nearly completely restored HIV reactivation. Moreover, G-MDSCs from HD, which inherently maintain high ARG1 expression, similarly showed a trend toward restricting HIV reactivation in allogeneic cocultures, supporting the notion that suppressive capacity reflects an intrinsic enzymatic property of G-MDSCs rather than an HIV-specific alteration. Collectively, these findings suggest that targeting L-arginine metabolism, either through L-arginine supplementation or treatment with an ARG1 inhibitor, could restore some T cell functions and lower the threshold for viral reactivation, potentially enhancing the efficacy of “shock and kill” strategies in PWH on ART.

Overall, our study reveals the persistence of elevated levels of MDSCs during ART, which retain their potent immunosuppressive traits and may contribute to the perpetuation of HIV reservoirs, thus providing a deeper understanding of the mechanisms underlying HIV-induced immunoregulation. Consequently, we propose that targeting MDSC-mediated immunosuppression could represent a promising avenue to eliminate HIV following the disruption of viral latency in ART individuals.

### Limitations of the study

One limitation of our study is its cross-sectional design of the MDSC characterization, which precludes establishing causal relationships between HIV infection and MDSC frequencies. Longitudinal studies are necessary to determine whether fluctuations in MDSC levels and immunosuppressive potential precede changes in HIV reservoir markers and clinical parameters, or vice versa.

## Methods

### Sex as a biological variable.

In this study, we used peripheral blood mononuclear cells (PBMCs) obtained from blood samples from PWH and non-HIV donors (HD). The PWH cohort was predominantly male, reflecting the epidemiology of HIV infection in our setting, whereas sex information was not available for HD. Therefore, sex-stratified analyses were not performed.

### Study samples.

Blood samples from 2 cohorts of PWH (*n* = 62) were collected at the HIV unit of the HUVH in Barcelona, Spain. These cohorts included PWH with detectable viremia (VIR; *n* = 13; HIV-1 RNA = 880–151,000 copies/mL, CD4^+^ T cell counts = 220–950 cells/μL) and PWH receiving suppressive ART (ART; *n* = 49, CD4^+^ T cell counts = 280–1730 cells/μL) with undetectable VL for 4–153 months. Blood samples from HD (*n* = 27) were obtained from the Blood and Tissue Bank in Barcelona, Spain (register no. C.0003590). Formalin-fixed and paraffin-embedded (FFPE) LN samples from PWH (VIR *n* = 4 and ART *n* = 1) were obtained from the Anatomical Pathology Department of HUVH. Detailed demographic and clinical information is provided in Supplemental Table 1.

### Cells and reagents.

The main reagents, manufacturers, and catalog numbers are listed in Supplemental Table 2. PBMCs were isolated by Ficoll-Paque density-gradient centrifugation and immediately cryopreserved in liquid nitrogen using RPMI 1640 containing 40% FBS and 10% dimethyl sulfoxide (DMSO). Whenever required, PBMCs were cultured in RPMI 1640 supplemented with 10% FBS, 100 U/mL penicillin, and 100 μg/mL streptomycin (R10 medium) at 37°C and 5% CO_2_. CD4^+^ T cells were isolated from PBMCs using the MagniSort Human CD4^+^ T Cell Enrichment Kit, with 2 consecutive rounds of separation to maximize cell purity.

### Flow cytometric phenotyping of MDSCs.

Antibodies, cell dyes and materials are listed in Supplemental Table 3 and 4. To identify MDSC subsets and determine their frequencies in HD (*n* = 11) and VIR (*n* = 13) and ART (*n* = 26) PWH (participants HD#1–11, VIR#1–13, and ART#1–26, Supplemental Table 1), PBMCs were stained for surface and intracellular markers and analyzed by flow cytometry. PBMCs were first stained with LIVE/DEAD Fixable Violet Dead Cell Stain for 20 minutes at room temperature (RT). After washing with PBS, cells were incubated for 20 minutes at RT in staining buffer (SB; PBS 3% FBS) containing anti-CD33-PerCP-Cy5.5, anti-CD11b-FITC, anti-CD3-PE-Cy7, anti-HLA-DR-PE-Dazzle594, anti-CD4-AF700, anti-CD15-BV605, and anti-CD14-V500 antibodies. Cells were then washed with SB and fixed/permeabilized using Fixation/Permeabilization Solution for 20 minutes at 4°C, followed by 2 washes with BD Perm/Wash buffer. Intracellular staining was subsequently performed with anti-ARG1-APC and anti-IDO-1-PE antibodies for 30 minutes at RT. Finally, cells were washed twice with BD Perm/Wash buffer, and fixed in 2% paraformaldehyde (PFA).

Samples were acquired on an LSRFortessa flow cytometer (BD Biosciences), and analyzed using the OMIQ platform (Dotmatics). All events within pregated live cells were concatenated for each group (HD, VIR, and ART) and clustering was performed using the Flow-Self Organizing Maps (FlowSOM) algorithm based on CD3, CD4, CD11b, CD33, HLA-DR, CD14, and CD15 expression. To visualize the resulting clusters, dimensionality reduction was performed using opt-SNE on a randomly selected subset of 5 × 10^5^ cells per group using the same marker set.

To evaluate the impact of cryopreservation on myeloid cell recovery, the total frequency of MDSCs (CD3^–^CD33^+^CD11b^+^HLA-DR^–^) was compared between fresh and cryopreserved PBMC samples from 3 ART PWH. A modest, negligible reduction in MDSC frequency was observed after thawing, indicating minimal impact of cryopreservation on downstream analyses (Supplemental Figure 9).

### Immunofluorescence and IHC of human LN samples.

Immunodetection of the HIV p24 protein and the MDSCs marker CD33, as well as CD8 and GzmB, was conducted separately on anatomically intact LN tissue preparations from VIR (*n* = 3) and ART (*n* = 1) PWH (participants VIR#14–16 and ART#50, Supplemental Table 1). FFPE tissue sections (3 μm) were subjected to deparaffinization, using decreasing ethanol concentrations, followed by hydration. Heat-induced epitope retrieval was performed by autoclaving at 120°C for 15 minutes in Tris-EDTA Buffer (pH 9) for p24-CD33 staining or in Citrate Buffer (pH 6) for CD8-GzmB staining. Sections were then permeabilized in 1X Tris-buffered saline (TBS) with 0.1% Triton X-100 and 1% BSA for 10 minutes, blocked with 1X TBS supplemented with 10% normal donkey serum and 1% BSA for 2 hours and incubated overnight at 4°C with primary antibodies against CD33 and p24, or CD8 and GzmB (all diluted in 1X TBS, 1% BSA). After washing, sections were incubated for 1 hour with Alexa Fluor-conjugated secondary antibodies: donkey anti-rabbit AF488, donkey anti-mouse AF647 and donkey anti-rat AF594. Nuclei were counterstained with DAPI, and slides were mounted with Fluoromount G. Sequential imaging was performed on an Olympus Spectral Confocal Microscope FV1000 (×20 and ×40 objectives). ImageJ 1.53c software (NIH) was used to perform image processing and analysis. For CD33/p24 and CD8/GzmB colocalization analysis, a binary mask was generated for each channel, and the “analyze particles” tool was used to quantify single- and double-stained cells across at least 10 B cell follicles per participant.

Immunohistochemical staining for CD33 and HLA-DR was performed on FFPE LN sections (3 μm) from VIR (*n* = 1) and ART (*n* = 1) PWH (participants VIR#17 and ART#50, Supplemental Table 1) using the Roche Ventana BenchMark ULTRA automated slide staining system (Ventana Medical Systems). Antigen retrieval was achieved with Ventana CC1 (cell conditioning solution) for 36 minutes for HLA-DR and 64 minutes for CD33. Sections were incubated with the primary antibodies: anti-HLA-DR (36 minutes) and anti-CD33 (32 minutes). Staining was visualized using the OptiView DAB IHC Detection Kit, and images were captured on an Olympus BX43 microscope. Primary, secondary antibodies and counterstains are listed in Supplemental Tables 5 and 6.

### Characterization of CD4^+^ Tregs and chronic immune activation.

CD4^+^ Tregs and markers of chronic immune activation in CD4^+^ and CD8^+^ T cells from HD (*n* = 10), VIR (*n* = 10) and ART (*n* = 9) individuals (participants HD#12–21, VIR#1, 4–10, 12, and 13, and ART#1–2, 5–8, 17, 22, and 25, Supplemental Table 1) were analyzed by flow cytometry. PBMCs were first stained with LIVE/DEAD Fixable Violet Dead Cell Stain for 20 minutes at RT. Following PBS washing, surface staining was performed in SB for 20 minutes at RT with anti-CD38-PerCP, anti-CD8-FITC, anti-CD3-PE-Cy7, anti-CD25-PE, anti-CD4-AF700, and anti-HLA-DR-SB600 antibodies. After washing with SB, cells were fixed/permeabilized using the FOXP3/Transcription Factor Fixation/Permeabilization Solution for 30 minutes at RT, followed by 2 washes with permeabilization buffer. Intracellular staining with anti-FOXP3-AF647 antibody was carried out for 30 minutes at RT, followed by a final wash and fixation in 2% PFA. Samples were acquired on a BD LSRFortessa flow cytometer, and analyzed using the OMIQ platform. Pregated live-cell events were concatenated by group (HD, VIR, and ART), and clustering was performed using the FlowSOM algorithm based on CD3, CD4, CD8, HLA-DR, CD38, CD25, and FOXP3 expression.

### Quantification of cell-associated HIV DNA and RNA in isolated CD4^+^ T cells by qPCR.

CD4^+^ T lymphocytes from VIR (*n* = 12) and ART (*n* = 16) PWH (participants VIR#2-13 and ART#1–3, 5, 7, 8, 13, 17, and 19–26, Supplemental Table 1) were enriched from PBMCs through negative selection, as described above, and used for both DNA and RNA quantification. For proviral HIV DNA quantification, 1 million CD4^+^ T cells were lysed overnight at 55°C in a Proteinase K–containing buffer, followed by heat inactivation at 95°C for 5 minutes. HIV DNA in the cell lysates was quantified by qPCR on a QuantStudio 5 Real-Time PCR System using primers and probes targeting the 1-LTR HIV region (Supplemental Table 7). The *CCR5* gene was used for cellular input normalization. Thermocycling conditions consisted of 50°C for 2 minutes, 95°C for 10 minutes, followed by 55 cycles of 95°C for 15 seconds, and 60°C for 1 minute. For viral RNA quantification, 1 million CD4^+^ T cells were subjected to total RNA extraction using the NZY Total RNA kit (Supplemental Table 8). Reverse transcription of RNA into cDNA was performed with SuperScript III Reverse Transcriptase and random primers. Total unspliced HIV transcripts were then quantified by qPCR using the same 1-LTR primer/probe set and cycling conditions. Absolute DNA and RNA copy numbers were determined using standard curves and normalized per million CD4^+^ T cells. HIV transcriptional activity was expressed as the ratio of HIV RNA copies to HIV DNA copies per million CD4^+^ T cells.

### Quantification of intact HIV DNA in isolated CD4^+^ T cells by Intact proviral DNA assay (IPDA).

To quantify intact HIV DNA within CD4^+^ T cells from ART PWH, IPDA was conducted on the same lysed CD4^+^ T cell samples used for total HIV DNA quantification by qPCR. Intact proviruses were measured on a QIAcuity One 2 plex System (Qiagen) using specific primers and probes targeting the HIV-1 Ψ *(psi)* and *env* regions, alongside an anti-hypermutant *env* probe. The human *RPP30* gene served as a reference for cellular input normalization. (Supplemental Table 9). Thermocycling conditions consisted of 95°C for 2 minutes, followed by 40 cycles of 95°C for 15 seconds and 60°C for 30 seconds. Intact proviral counts were corrected for DNA shearing and expressed as copies per million of CD4^+^ T cells. Strong concordance between LTR-based qPCR and IPDA-derived total HIV DNA was observed (Supplemental Figure 10), supporting the robustness of both assays.

### Ex vivo viral reactivation of latently HIV-infected CD4^+^ T cells and coculture with G-MDSCs.

CD4^+^ T lymphocytes from HD (*n* = 6) and ART PWH (*n* = 31) (participants HD#22–27 and ART#10–12, 14, 16–19, and 27–49, Supplemental Table 1) were isolated as described above. CD4^+^ T cells were then cultured at a cellular density of 1 × 10^6^ cells/mL in R10 medium containing 10 μM Q-VD-OPh, 1 μM Raltegravir, 1 μM Darunavir and 1 μM Nevirapine for 2 hours at 37°C and 5% CO_2_. After this incubation, CD4^+^ T lymphocytes were either left untouched or stimulated with 81 nM phorbol 12-myristate 13-acetate (PMA) and 1 μM ionomycin for 20–22 hours to induce viral reactivation. On the next day, a minimum of 100 M of PBMCs from the same ART individual or an HD were stained, and G-MDSCs (CD3^–^CD33^mid^CD11b^+^HLA-DR^–^CD14^–^) were sorted through Fluorescence-Activated Cell Sorting (FACS) using a FACSAria Cell Sorter (BD Biosciences) or an Aurora CS Flow Cytometer (Cytek Biosciences). The staining panel included LIVE/DEAD Fixable Aqua Stain, anti-CD33-PerCP-Cy5.5, anti-CD11b-FITC, anti-CD14-APC-H7, anti-CD3-AF700 or anti-CD3-PE-Cy7, and anti-HLA-DR-BV421 antibodies. Sorted G-MDSC purity consistently exceeded 95%. G-MDSCs were cocultured with reactivated CD4^+^ T cells in round-bottom 96-well plates at a 1:1 ratio overnight at 37°C and 5% CO_2_. For experiments assessing intact HIV DNA, G-MDSCs:CD4^+^ T cell ratios ranged from 1:1 to 1:3, depending on cell availability. Where indicated, cocultures were supplemented with 1 mM Nω-Hydroxy-nor-L-arginine monoacetate (nor-NOHA). Transwell experiments were performed using Corning HTS Transwell 96-well permeable supports to prevent direct cell-cell contact.

### Functional analysis of G-MDSCs activity.

The immunosuppressive effects of G-MDSCs on HIV reactivation and CD4^+^ T cell activation were assessed by flow cytometry. Viral reactivation was quantified by measuring p24 levels within CD4^+^ T cells, and CD4^+^ T cell activation was determined based on the surface expression of the early activation marker CD69. Three distinct antibody panels were used depending on the cytometer employed for sample acquisition: FACSCalibur (BD Biosciences), BD LSRFortessa, or Aurora CS Flow Cytometer. For the BD FACSCalibur, the panel comprised LIVE/DEAD Fixable Far Red Dead Cell Stain, anti-CD3-PerCP and anti-p24-PE antibodies. The BD LSRFortessa panel included: LIVE/DEAD Fixable Aqua Dead Cell Stain, anti-CD33-PerCP-Cy5.5, anti-CD3-PE-Cy7 and anti-CD69-PE-CF594 antibodies. For the Aurora CS, we used the following panel: Fixable Far Red Dead Cell Stain, anti-CD3-AF700, anti-CD33-PerCP-Cy5.5, anti-CD69-PE-CF594 and anti-p24-PE antibodies. Cell permeabilization/fixation for intracellular p24 staining were conducted using the BD Cytofix/Cytoperm kit. Cells were incubated with anti-p24 antibody for 30 minutes on ice, followed by 30 minutes at RT. Lastly, cells were fixed with 2% PFA, and acquired on their respective flow cytometers. The acquired data were analyzed using FlowJo v10 software (Tree Star Inc.).

To assess the impact of G-MDSCs on the HIV reservoir, CD4^+^ T cells were reisolated following coculture using the CD4^+^ T cell enrichment method described above. Purified CD4^+^ T cells were lysed, and intact HIV DNA was quantified by IPDA, both performed as previously described.

### Reagents and materials availability.

All antibodies and chemical reagents used in this study are commercially available and validated by their respective manufacturers. Detailed information regarding all antibodies, buffers, kits, oligonucleotides, and other materials is consolidated in Supplemental Tables 2–9.

### Statistics.

Graphing and statistical analyses were performed using GraphPad Prism 8.3.0 software (GraphPad Software). Statistical differences between experimental groups were determined using 2-sided Mann-Whitney *U* tests or Kruskal-Wallis tests followed by Dunn’s post hoc correction. *P* < 0.05 was considered statistically significant. Volcano plots, generated using OMIQ software, represent statistical comparisons conducted via the edgeR method (2-sided), with significance determined at *P* < 0.05. Group sizes (*n*) and detailed statistical information for each experiment are specified in the respective figure legends.

### Study approval.

The study protocols received ethical approval from the IRB at HUVH [PR(AG)270/2015, PR(AG)582/2020]. All participants were adults who provided written informed consent prior to inclusion and did not receive monetary compensation. The anonymity and untraceability of the collected samples were ensured.

### Data availability.

All data associated with this study are available within the main text or the Supplemental Information. Source data are provided with this paper in the Supporting Data Values file. Any other requested data related to samples from HD and PWH is restricted by ethical and privacy considerations. Access can be obtained after IRB approval of the formal data-sharing agreement in a process that can last up to 24 weeks.

## Author contributions

AGC and MJB designed, directed, and interpreted experiments. AGC, JGE, IMG, ABM, and JC performed the experiments and analyzed the data. MG contributed to experimental design and data interpretation. JC, JN, AC, JB, PS, and VF were responsible for participant recruitment and clinical data collection. AGC and MJB wrote the initial manuscript, and all authors contributed to its editing.

## Conflict of interest

The authors have declared that no conflict of interest exists.

## Funding support

- Agencia Estatal de Investigación (projects PID2021-123321OB-I00 and PID2024-155881OB-100 funded by MCIN/AEI/10.13039/501100011033/FEDER, UE) to MJB.
- The Spanish “Ministerio de Economía y Competitividad, Instituto de Salud Carlos III” (ISCIII, PI20/00160) to MG.
- Gilead fellowships (GLD21-00049, GLD22/00152 and GLD24/00014) to MJB and MG.
- Miguel Servet program of the ISCIII (CP17/00179 and CPII22/00005) (MJB).
- PhD fellowship from “la Caixa” Foundation (LCF/BQ/DI20/11780014) (AGC).

## Supplementary Material

Supplemental data

Supporting data values

## Version 1 07/21/2026

In-Press Preview

## Version 2 08/24/2026

Electronic publication
