# Supplementary material for: Granulocytic myeloid–derived suppressor cells sustain HIV reservoirs by inhibiting viral reactivation via arginase 1–mediated mechanisms
Source: JCI Insight. 2026 Jul 21;11(16):e202628. doi: 10.1172/jci.insight.202628 (PMC13502176; doi:10.1172/jci.insight.202628)
Supplement: Supplementary file 1 [file jciinsight-11-202628-s328.pdf]

Supplemental Figure 1

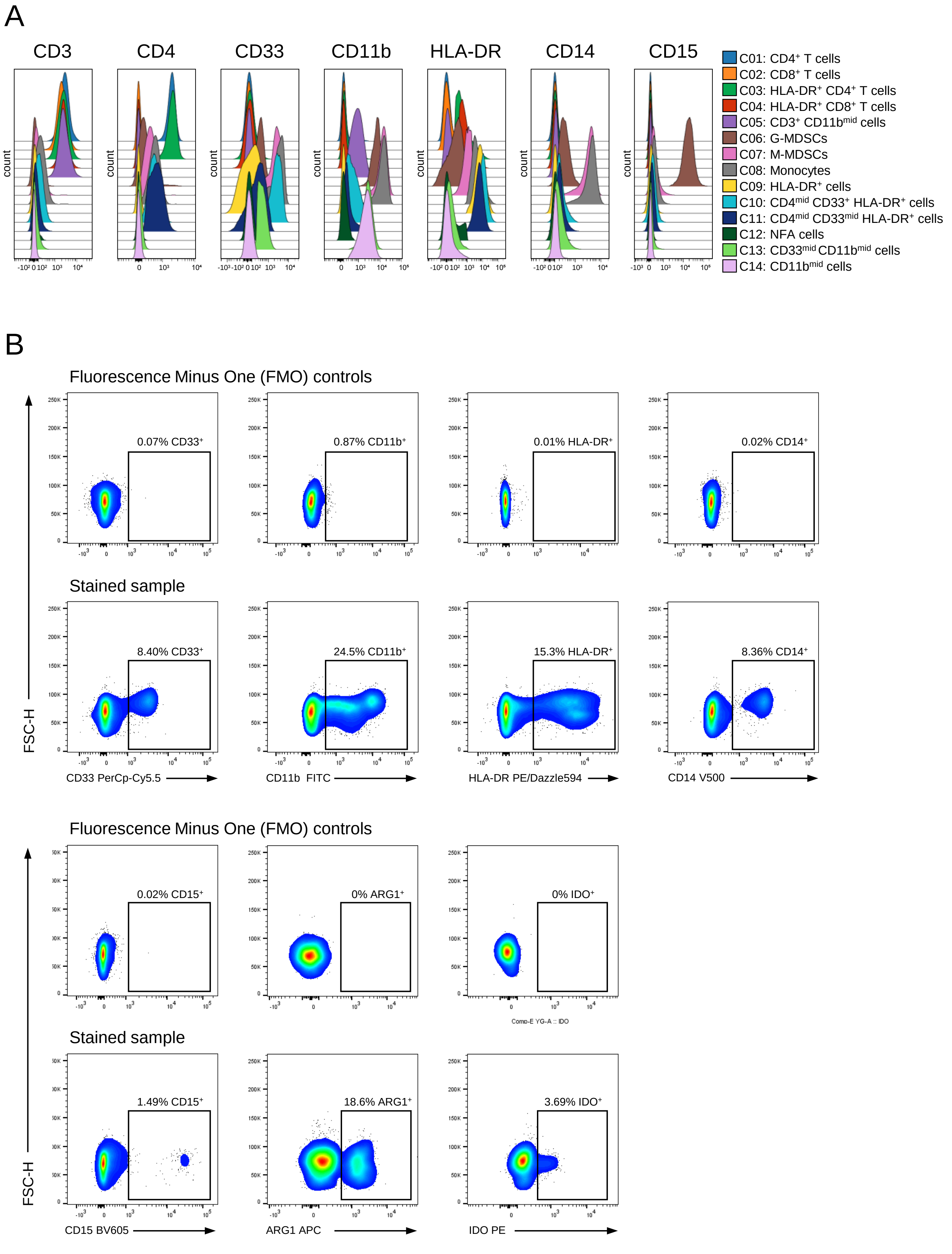

Supplemental Figure 1. Expression of markers used in the phenotypic characterization of MDSC subsets. Related to Figures 1 and 2.

(A) Histograms showing the Mean Fluorescence Intensity (MFI) of expression of the markers within each of the fourteen clusters, each represented in different colours. All cohorts are included (n_{HD} = 11, n_{VIR} = 13 and n_{ART} = 26).

(B) Fluorescence Minus One (FMO) controls of the markers. FMOs for CD33, CD11b, HLA-DR, CD14 and CD15 are gated within live cells. FMOs for ARG1 and IDO are gated within $CD3^-$ $CD33^+$ $CD11b^+$ HLA-DR^{low} live cells.

Supplemental Figure 2

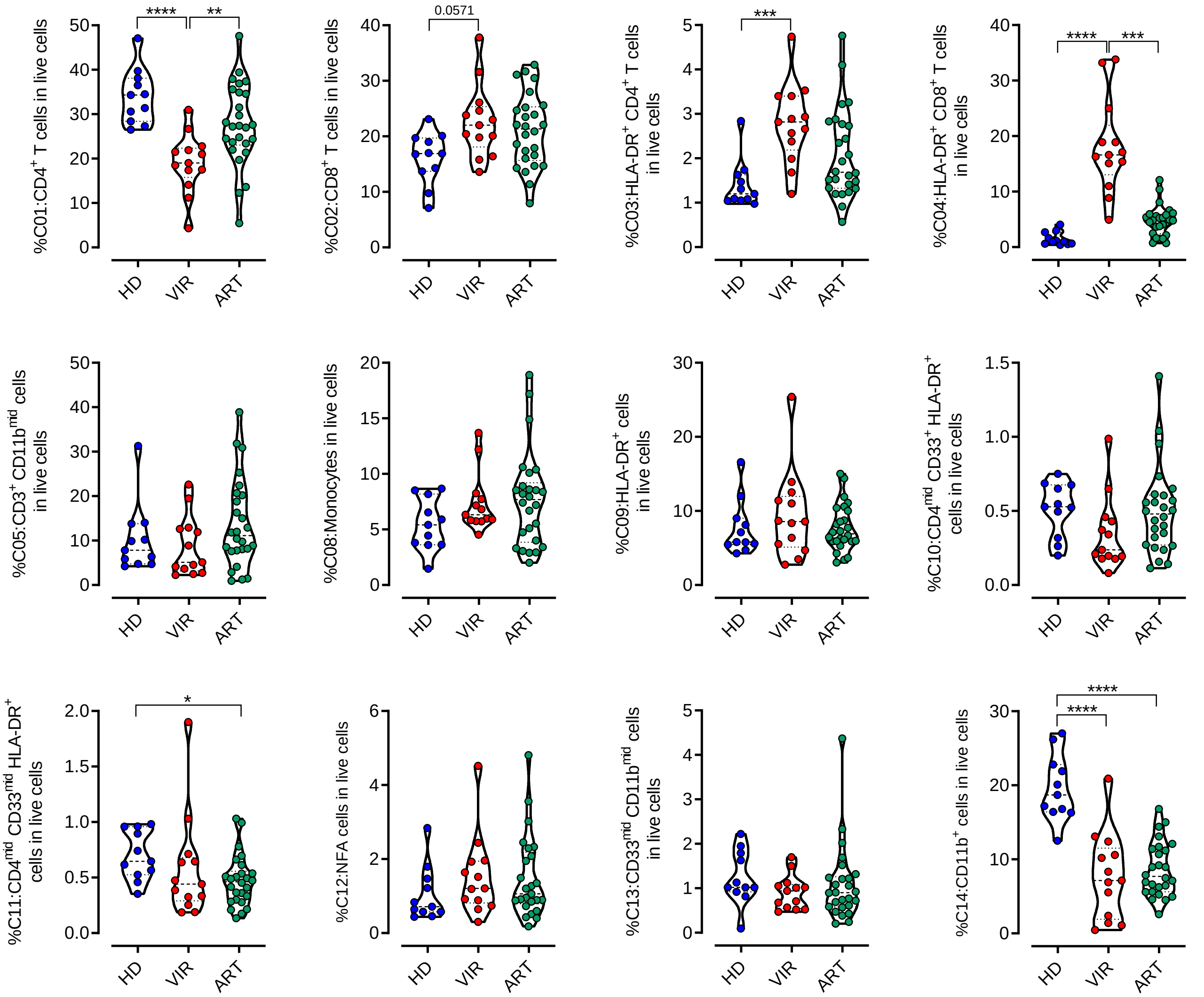

Supplemental Figure 2. Phenotypic characterization of PBMCs from PWH and HD. Violin plots displaying the frequency of the twelve remaining identified clusters within total live PBMCs in HD (*n* = 11), VIR (*n* = 13) and ART (*n* = 26) individuals. Median values with quartiles are represented, and statistical comparisons were conducted using the two-sided Kruskal-Wallis test with Dunn's post hoc correction. Significance levels are indicated as *p < 0.05, **p < 0.01, ***p < 0.001 and ****p < 0.0001. Related to Figure 1. Source data are provided as a Source Data file.

Supplemental Figure 3

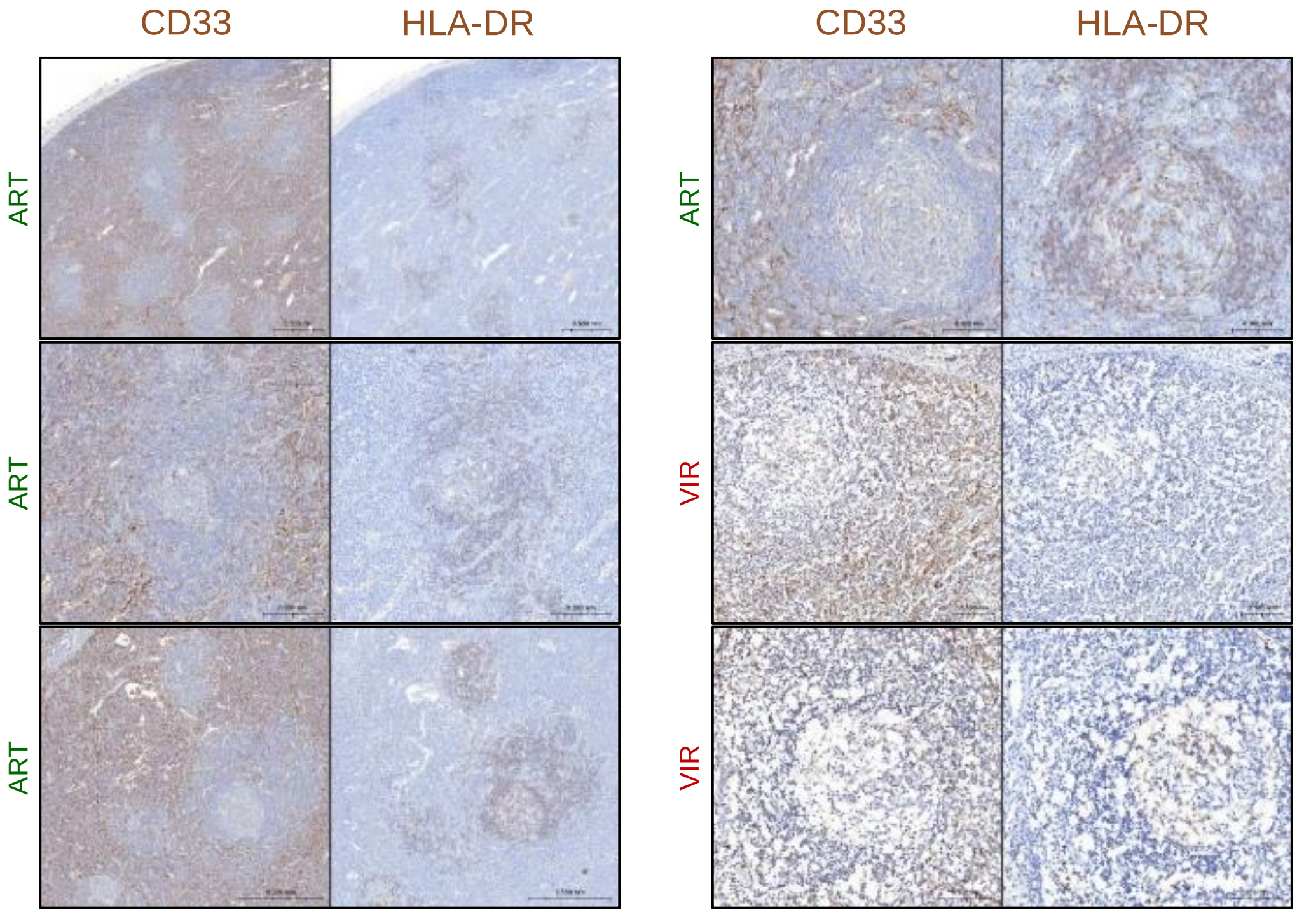

Supplemental Figure 3. Representative immunohistochemistry images of lymph node sections from two PWH (n_{VIR} = 1 and n_{ART} = 1) illustrating CD33/HLA-DR expression patterns (×20 and ×40 magnification). Most $CD33^{+}$ cells lack HLA-DR expression, confirming their identity as MDSCs. Related to Figure 2.

Supplemental Figure 4

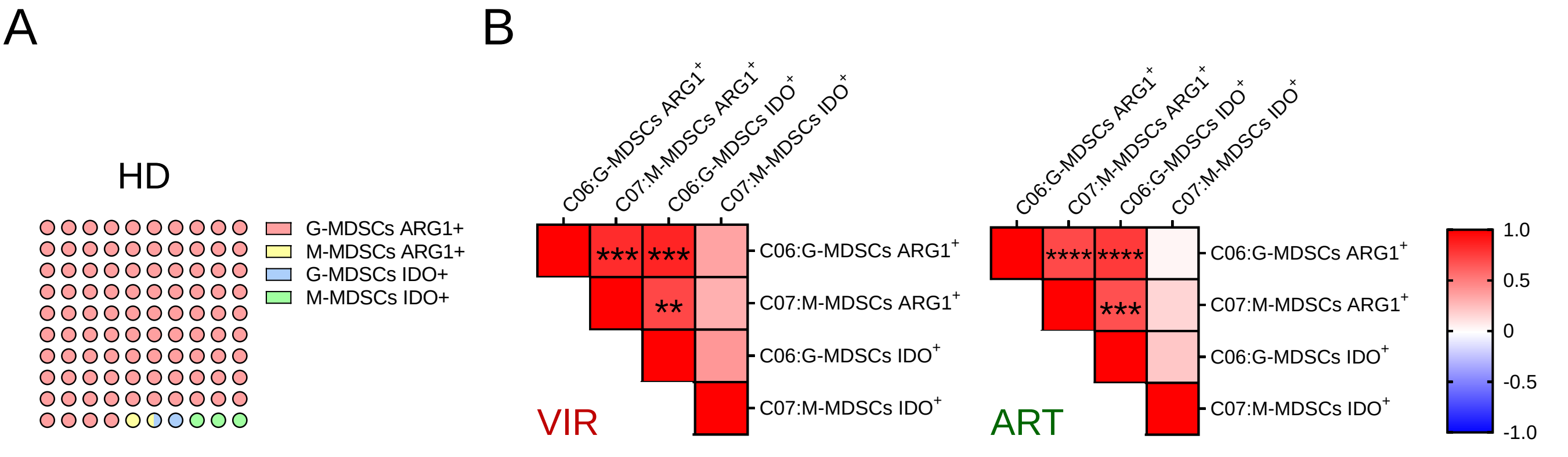

Supplemental Figure 4. Elevated frequencies of MDSCs with enhanced immunosuppressive capacities in PWH. Related to Figure 3.

(A) Dot plot representing the proportion of ARG1+ and IDO+ subsets of G-MDSCs (C06) and M-MDSCs (C07) from total MDSCs expressing ARG1 and/or IDO in the HD cohort (*n* = 11).

(B) Correlations between ARG1+ and IDO+ subsets of G-MDSCs (C06) and M-MDSCs (C07) within the VIR (*n* = 13) and ART (*n* = 26) cohorts. Correlations were assessed using Spearman analysis with significance denoted as **p < 0.01, ***p < 0.001 and ****p < 0.0001.

Source data are provided as a Source Data file.

Supplemental Figure 5

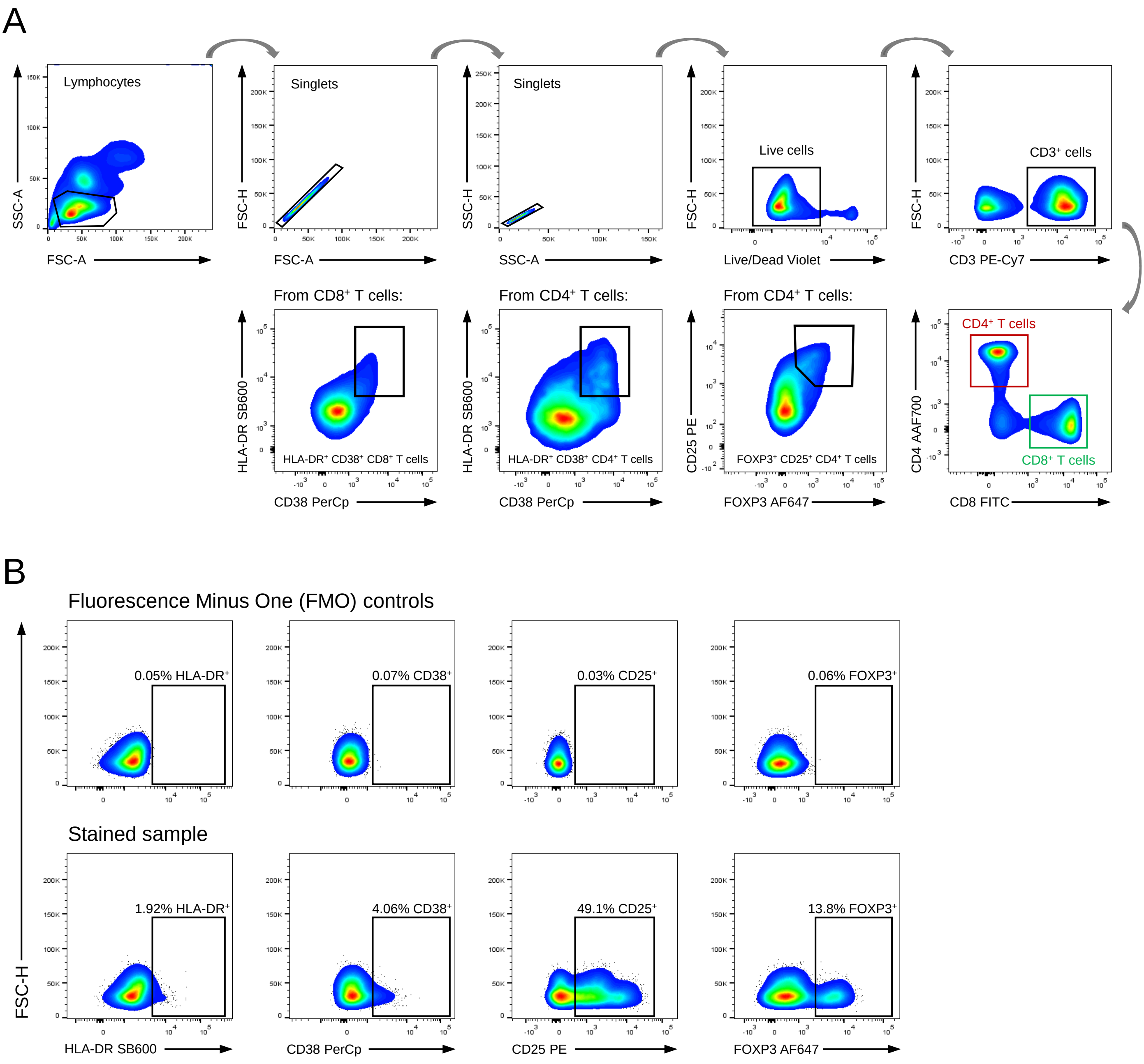

Supplemental Figure 5. Regulatory T cells (T_{regs}) and chronic immune activation in the studied cohorts. Related to Figure 4.

(A) Gating strategy used for the analysis of regulatory T cells (T_{regs}) and chronic immune activation in $CD4^+$ and $CD8^+$ T cells. $CD4^+$ and $CD8^+$ T cells were defined as lymphocyte-shaped live $CD3^+CD4^+CD8^-$ and $CD3^+CD4^-CD8^+$ populations, respectively. T_{regs} were identified as $CD4^+$ T cells co-expressing CD25 and FOXP3. Chronic immune activation was assessed by the co-expression of HLA-DR and CD38 on $CD3^+$ T cells.

(B) Fluorescence Minus One (FMO) controls for the markers used to identify chronically activated T cells and T_{regs}. FMOs for HLA-DR and CD38 were gated within live $CD3^+$ cells, while FMOs for CD25 and FOXP3 were gated within live $CD3^+CD4^+$ cells.

Supplemental Figure 6

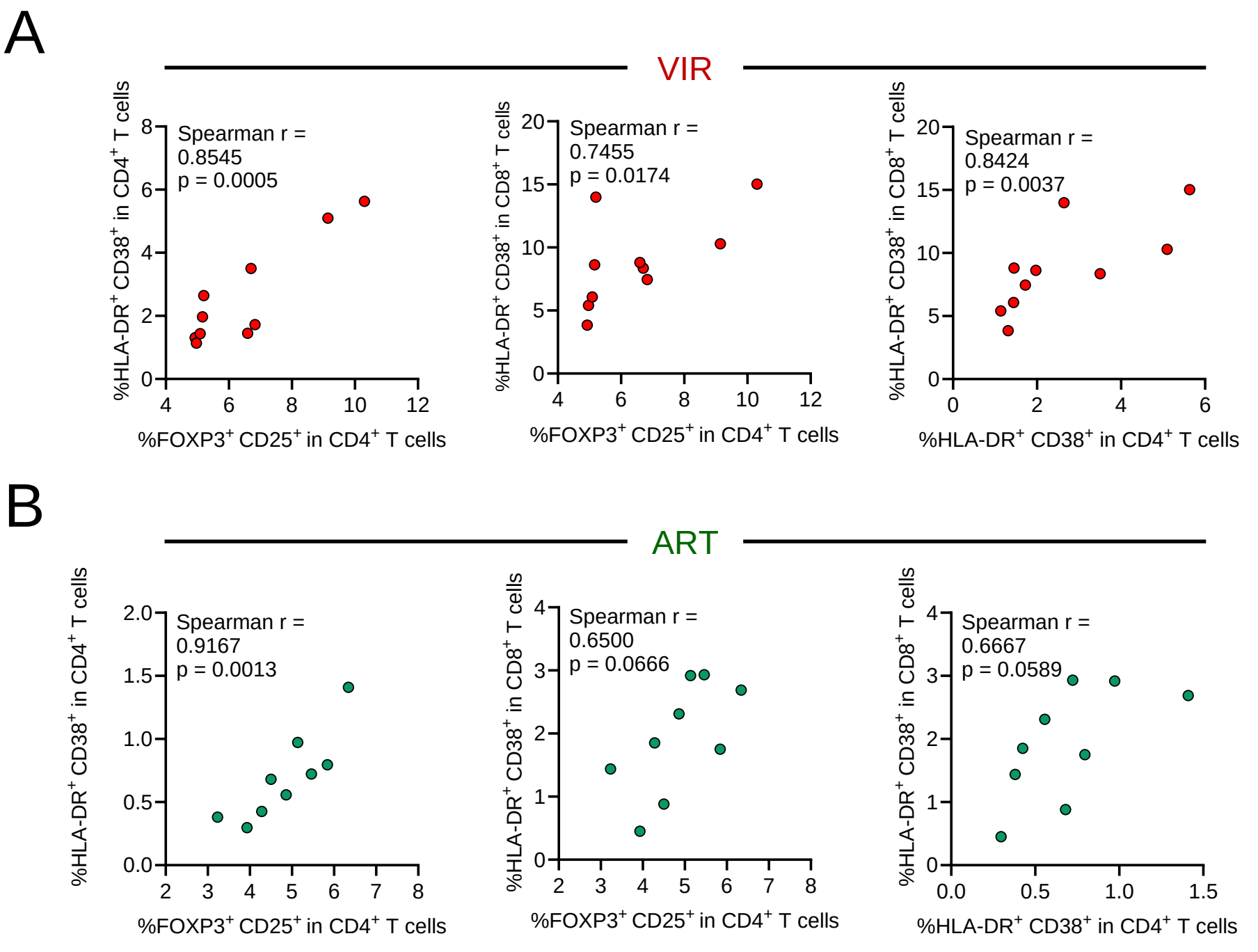

Supplemental Figure 6. Correlations between regulatory T cells (T_{regs}) and chronic immune activation in PWH. Correlation analyses between T_{reg} frequencies and chronic immune activation, in the VIR (*n* = 10) (A) and ART (*n* = 9) (B) cohorts. Correlations were assessed using Spearman analysis. Related to Figure 4. Source data are provided as a Source Data file.

Supplemental Figure 7

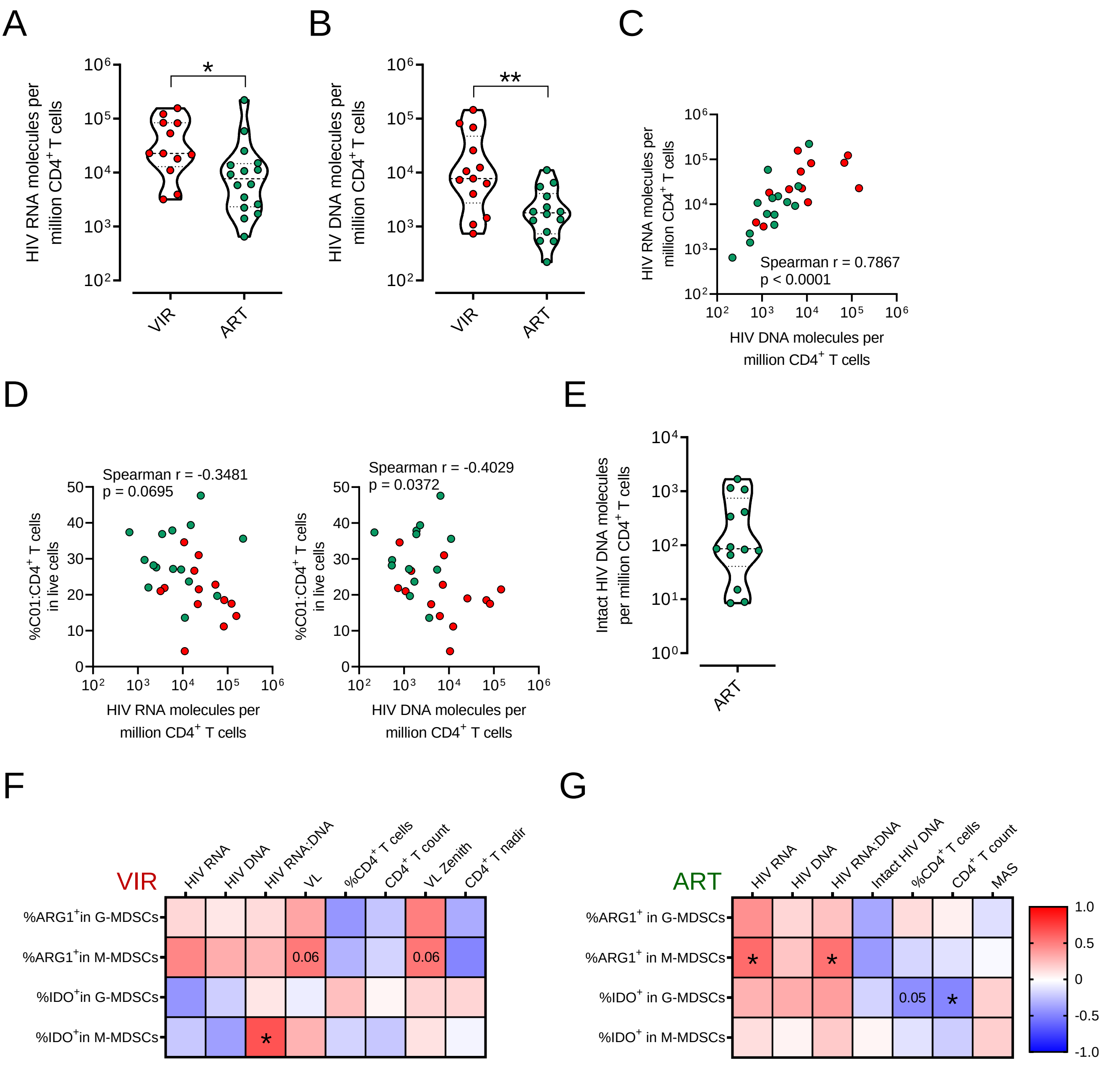

Supplemental Figure 7. HIV reservoir parameters in PWH. Related to Figure 5.

(A-B) Violin plots illustrating HIV RNA (A) and HIV DNA (B) copies within one million of isolated $CD4^+$ T cells from VIR and ART individuals. Median values with quartiles are represented, and statistical comparisons were performed using the two-sided Mann-Whitney test. Significance levels are denoted as $*p<0.05$ and $**p<0.01$.

(C) Correlation between HIV RNA and HIV DNA in PWH.

(D) Correlations between the percentage of $CD4^+$ T cells (C01) and HIV RNA and HIV DNA in PWH.

(E) Violin plot showing the levels of intact HIV DNA molecules per million of isolated $CD4^+$ T cells from ART PWH.

(F) Correlations between the expression of ARG1 and IDO in G-MDSCs (C06) and M-MDSCs (C07) and HIV RNA (n = 12) and HIV DNA (n = 13) within $CD4^+$ T cells, HIV RNA:DNA ratio (n = 12), Viral Load (VL) (n = 13), frequency of $CD4^+$ T cells (C01) (n = 13), $CD4^+$ T-cell count (n = 13), VL Zenith (n = 13) and nadir $CD4^+$ T-cell count (n = 13) in the VIR cohort.

(G) Correlations between ARG1 and IDO expression in both MDSC subsets and HIV RNA (n = 16) and HIV DNA (n = 14) within $CD4^+$ T cells, HIV RNA:DNA ratio (n = 14), intact HIV DNA in $CD4^+$ T cells (n = 13), frequency of $CD4^+$ T cells (C01) (n = 26), $CD4^+$ T-cell count (n = 26) and months on suppressive ART (MAS) (n = 26) in the ART cohort.

Correlations were assessed using Spearman analysis with significance denoted as $*p < 0.05$. Source data are provided as a Source Data file.

Supplemental Figure 8

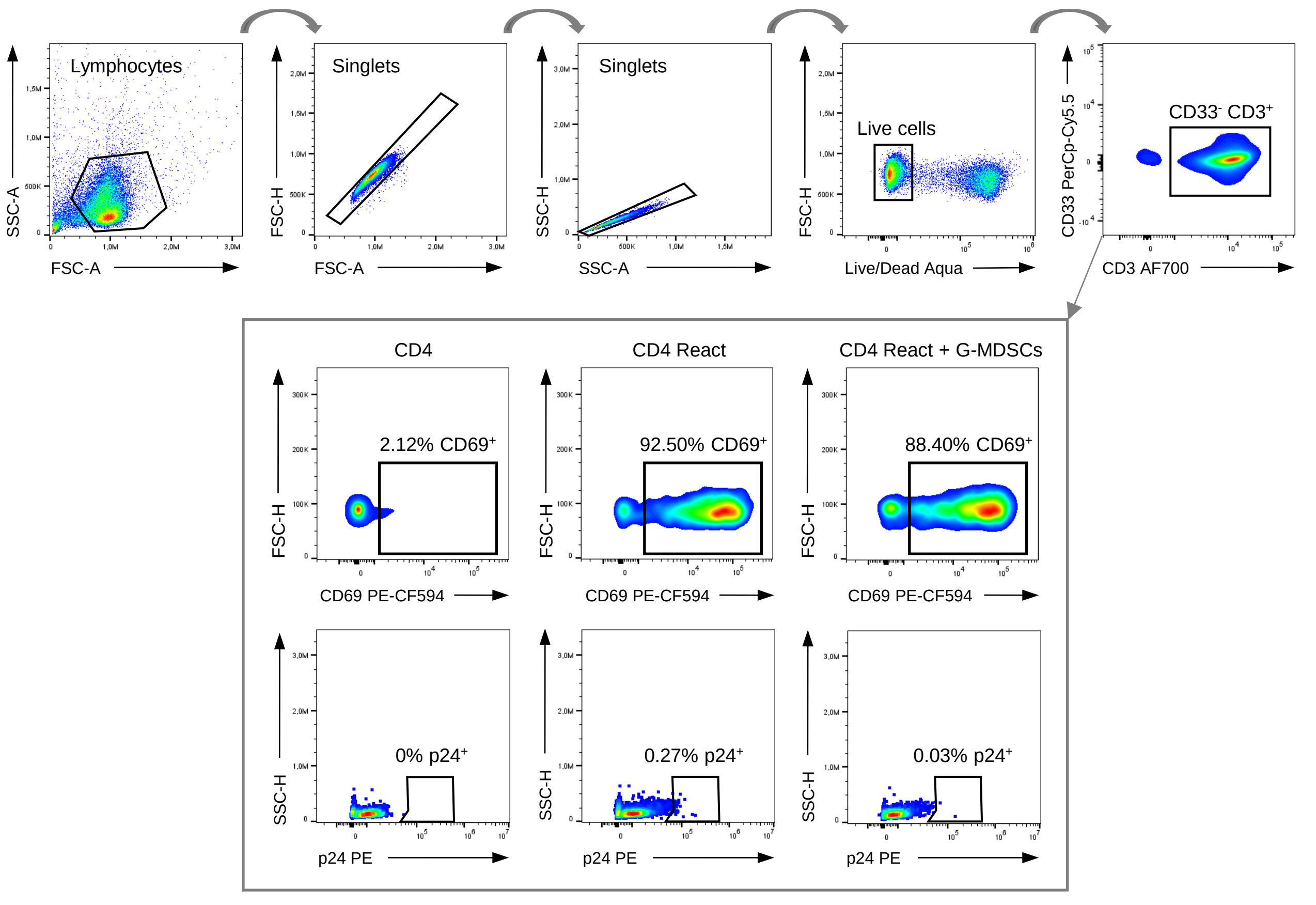

Supplemental Figure 8. Gating strategy used for the detection of CD69 and p24 in CD4⁺ T cells after ex vivo reactivation and T-cell and G-MDSCs co-culture. CD4⁺ T cells were identified as live CD3⁺ CD33⁻ cells. Related to Figure 6.

Supplemental Figure 9

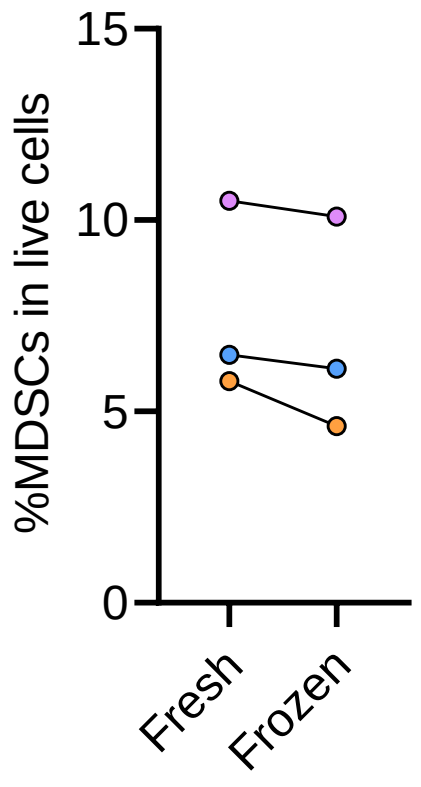

Supplemental Figure 9. Viability of MDSCs from fresh and cryopreserved PBMCs. Frequencies of MDSCs ($CD3^{-}CD33^{+}CD11b^{+}HLA\text{-}DR^{-}$) in live PBMCs from paired fresh and cryopreserved PBMC samples from three ART PWH.

Supplemental Figure 10

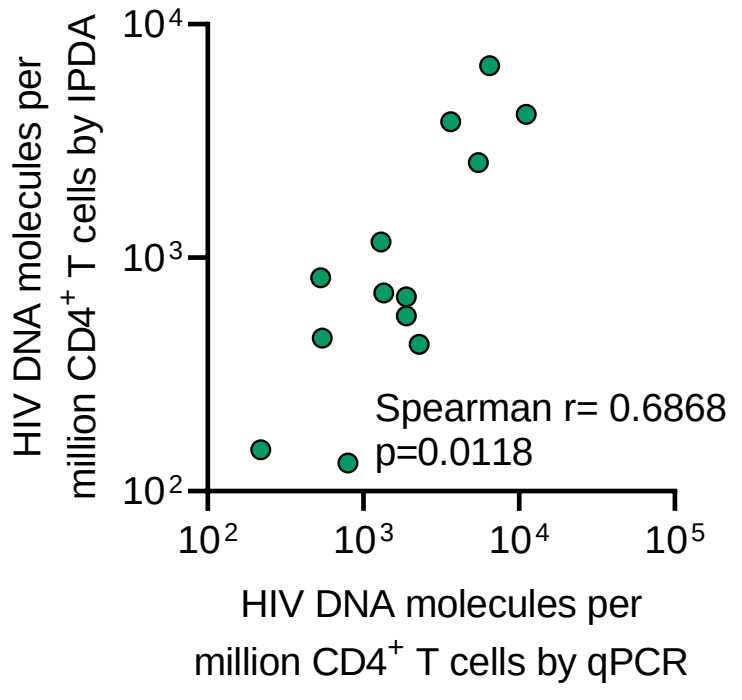

Supplemental Figure 10. Correlation between LTR-based qPCR and IPDA-derived total HIV DNA measurements. Correlation analysis between total HIV DNA levels quantified by 1-LTR-based qPCR and IPDA in $CD4^+$ T cells from ART PWH. Spearman correlation coefficient and p value are indicated.

Supplemental Table 1

#ID	Age (years)	Gender	Time since HIV diagnosis (months)	$CD4^+$ T-cell count (cells/µl)	%$CD4^+$ T cells	Viral Load (copies/ml)	Time on ART with VL suppressed (months)	ART regimen
HD-1	ND	ND	NA	ND	ND	NA	NA	NA
HD-2	ND	ND	NA	ND	ND	NA	NA	NA
HD-3	ND	ND	NA	ND	ND	NA	NA	NA
HD-4	ND	ND	NA	ND	ND	NA	NA	NA
HD-5	ND	ND	NA	ND	ND	NA	NA	NA
HD-6	ND	ND	NA	ND	ND	NA	NA	NA
HD-7	ND	ND	NA	ND	ND	NA	NA	NA
HD-8	ND	ND	NA	ND	ND	NA	NA	NA
HD-9	ND	ND	NA	ND	ND	NA	NA	NA
HD-10	ND	ND	NA	ND	ND	NA	NA	NA
HD-11	ND	ND	NA	ND	ND	NA	NA	NA
HD-12	ND	ND	NA	ND	ND	NA	NA	NA
HD-13	ND	ND	NA	ND	ND	NA	NA	NA
HD-14	ND	ND	NA	ND	ND	NA	NA	NA
HD-15	ND	ND	NA	ND	ND	NA	NA	NA
HD-16	ND	ND	NA	ND	ND	NA	NA	NA
HD-17	ND	ND	NA	ND	ND	NA	NA	NA
HD-18	ND	ND	NA	ND	ND	NA	NA	NA
HD-19	ND	ND	NA	ND	ND	NA	NA	NA
HD-20	ND	ND	NA	ND	ND	NA	NA	NA
HD-21	ND	ND	NA	ND	ND	NA	NA	NA
HD-22	ND	ND	NA	ND	ND	NA	NA	NA
HD-23	ND	ND	NA	ND	ND	NA	NA	NA
HD-24	ND	ND	NA	ND	ND	NA	NA	NA
HD-25	ND	ND	NA	ND	ND	NA	NA	NA
HD-26	ND	ND	NA	ND	ND	NA	NA	NA
HD-27	ND	ND	NA	ND	ND	NA	NA	NA
VIR-1	49	M	260	510	36.9	90100	NA	UNT
VIR-2	19	M	*4 days	460	32.0	52900	NA	UNT
VIR-3	44	M	*14 days	950	37.4	45800	NA	UNT
VIR-4	40	M	26	560	29.9	3510	NA	UNT
VIR-5	23	M	8	420	30.5	74500	NA	UNT
VIR-6	52	F	203	330	8.9	79200	NA	UNT
VIR-7	36	M	2	890	33.9	17500	NA	UNT
VIR-8	46	M	49	670	25.0	26600	NA	UNT
VIR-9	53	M	336	220	15.3	151000	NA	UNT
VIR-10	45	M	219	360	19.8	880	NA	UNT
VIR-11	53	M	213	510	21.9	74900	NA	UNT
VIR-12	39	M	*4 days	370	16.8	37700	NA	UNT
VIR-13	46	M	68	480	19.8	40700	NA	UNT
VIR-14	57	M	1	61	8.7	5000000	NA	UNT
VIR-15	38	F	201	303	23.3	96000	NA	UNT
VIR-16	47	M	42	400	20.0	129987	NA	UNT
VIR-17	38	M	81	144	8.0	6700	NA	UNT
ART-1	29	M	11	280	22.5	<50	4	ABC+3TC+DTG
ART-2	32	M	57	430	24.7	<50	5	TAF+FTC+EVG/COB
ART-3	37	M	27	600	31.2	<50	18	ABC+3TC+ATV/RTV
ART-4	32	M	40	690	37.4	<50	21	TAF+FTC+EVG/COB
ART-5	39	M	30	910	34.0	<50	22	TAF+FTC+EVG/COB
ART-6	50	M	31	840	35.8	<50	23	TAF+FTC+EVG/COB
ART-7	57	F	31	1290	61.8	<50	27	TDF+FTC+RPV
ART-8	37	M	57	990	42.6	<50	34	TAF+FTC+DRV/COB
ART-9	41	M	181	280	10.9	<50	36	TAF+FTC+BIC
ART-10	37	M	47	790	27.6	<50	42	TAF+FTC+BIC
ART-11	32	M	45	570	40.8	<50	45	3TC+DTG
ART-12	34	M	145	540	24.3	<50	46	TAF+FTC+DRV/COB
ART-13	61	M	293	380	22.4	<50	48	3TC+DRV/COB
ART-14	42	F	51	560	31.3	<50	50	TAF+FTC+DRV/COB
ART-15	33	M	68	950	42.3	<50	52	TAF+FTC+BIC

#ID	Age (years)	Gender	Time since HIV diagnosis (months)	$CD4^+$ T-cell count (cells/µl)	$\%CD4^+$ T cells	Viral Load (copies/ml)	Time on ART with VL suppressed (months)	ART regimen
ART-16	35	M	121	800	26.9	<50	56	TAF+FTC+BIC
ART-17	32	M	160	1240	44.0	<50	67	TAF+FTC+BIC
ART-18	56	F	308	1730	51.7	<50	70	RPV+DTG
ART-19	39	F	79	1560	51.1	<50	71	TDF+FTC+EFV
ART-20	35	F	214	1000	37.0	<50	77	TDF+FTC+RPV
ART-21	52	F	90	710	38.3	<50	85	TAF+FTC+DRV/COB
ART-22	31	M	96	840	39.0	<50	89	TDF+FTC+EFV
ART-23	42	M	109	580	39.2	<50	100	TDF+FTC+ATV/COB
ART-24	59	M	135	810	44.3	<50	104	ABC+3TC+EFV
ART-25	49	F	268	1610	46.4	<50	129	TDF+FTC+NVP
ART-26	70	F	403	680	23.5	<50	153	RPV+DRV/COB
ART-27	52	F	64	640	21.6	<50	47	TAF+FTC+BIC
ART-28	47	M	79	940	45.4	59	52	3TC+DTG
ART-29	48	M	51	430	28.8	<50	47	TAF+FTC+BIC
ART-30	52	M	85	400	25.0	<50	53	TAF+FTC+DRV/COB
ART-31	27	F	80	1210	45.9	<50	29	TAF+FTC+EVG/COB
ART-32	29	F	84	550	26.8	<50	84	ABC+3TC+RTG
ART-33	37	F	183	1300	37.7	<50	80	ABC+3TC+RPV
ART-34	41	M	52	680	36.0	<50	43	TAF+FTC+EVG/COB
ART-35	28	M	31	1030	40.0	<50	27	3TC+DTG
ART-36	61	F	70	1050	57.1	<50	62	TAF+FTC+RPV
ART-37	40	M	95	1120	43.0	<50	70	TAF+FTC+DRV/COB
ART-38	47	M	312	340	19.0	<50	80	TAF+FTC+DRV/COB
ART-39	46	F	328	1250	39.0	<50	76	ABC+3TC+DTG
ART-40	82	M	141	550	30.4	<50	54	TAF+FTC+BIC
ART-41	34	M	129	670	27.7	<50	74	ABC+3TC+DTG
ART-42	45	M	226	510	34.6	<50	123	TDF+FTC+RPV
ART-43	46	M	65	1480	39.0	<50	63	TAF+FTC+RTG
ART-44	51	M	92	770	30.0	<50	88	ABC+3TC+RPV
ART-45	63	M	285	290	13.6	<50	55	TAF+FTC+BIC
ART-46	62	M	386	600	26.0	<50	17	COB+DRV+DTG
ART-47	24	F	25	440	19.9	<50	22	ABC+3TC+DTG
ART-48	34	M	94	580	31.6	<50	59	TAF+FTC+DRV/COB
ART-49	37	M	72	920	39.3	<50	66	TAF+FTC+DRV/COB
ART-50	55	M	96	318	21.2	<50	38	LPV/RTV

Supplemental Table 1. Clinical data of PWH included in the study. This table summarizes the demographic and clinical characteristics of study participants, including the number of individuals per group, age, gender, time since HIV diagnosis, $CD4^+$ T-cell count and percentage, plasma viral load, duration of ART with sustained viral suppression, ART regimen. PWH: people with HIV; VL: viral load; ART: antiretroviral therapy; ND: Not Determined; NA: Not Applicable; M: male; F: female; UNT: untreated; ABC: abacavir; 3TC: lamivudine; DTG: dolutegravir; TAF: tenofovir alafenamida; FTC: emtricitabine; EVG: elvitegravir; COB: cobicistat; ATV: atazanavir; RTV: Ritonavir; TDF: tenofovir; RPV: Rilpivirine; DRV: darunavir; BIC: Bictegravir; EFV: efavirenz; NVP: nevirapine; LPV: Lopinavir. Related to Figures 1, 2, 3, 4, 5 and 6.

Supplemental Table 2

Reagent / Material	Manufacturer	Catalog No.
Lymphoprep	STEMCELL Technologies	7861
RPMI 1640 Medium	Life Technologies	12004997
Fetal Bovine Serum (FBS)	Life Technologies	A5256701
Dimethyl Sulfoxide (DMSO)	Fisher Scientific	10103483
Penicillin/Streptomycin	Labclinics	PS-B
MagniSort Human $CD4^+$ T Cell Enrichment Kit	Invitrogen	8804-6811-74
Q-VD-OPh	Selleckchem	S7311
Raltegravir	NIH AIDS Reagent Program	0980
Darunavir	NIH AIDS Reagent Program	0989
Nevirapine	Sigma-Aldrich	SML0097
Phorbol 12-myristate 13-acetate (PMA)	Abcam	ab120297
Ionomycin	Abcam	ab120370
Nω-Hydroxy-nor-L-arginine monoacetate (nor-NOHA)	ChemoSapiens	FH49048
Corning® HTS Transwell® 96-well permeable supports	Sigma-Aldrich	CLS3381

Supplemental Table 2. Reagents and Materials for PBMC Processing, Cell Enrichment and *Ex Vivo* Culture.

Supplemental Table 3

Marker	Fluorochrome	Clone	Manufacturer	Catalog No.
Viability (LIVE/DEAD)	Fixable Violet	N/A	Invitrogen	L34966
Viability (LIVE/DEAD)	Fixable Aqua	N/A	Invitrogen	L34957
Viability (LIVE/DEAD)	Fixable Far Red	N/A	Invitrogen	L34974
CD3	PE-Cy7	SK7	BD Biosciences	557851
CD3	AF700	SK7	BioLegend	344822
CD3	PerCP	SK7	BD Biosciences	345766
CD4	AF700	RPA-T4	BD Biosciences	557922
CD8	FITC	HIT8a	BD Biosciences	560960
CD11b	FITC	M1/70	BioLegend	101205
CD14	V500	MφP9	BD Biosciences	562693
CD14	APC-H7	MφP9	BD Biosciences	560180
CD15	BV605	W6D3	BD Biosciences	562980
CD25	PE	M-A251	BioLegend	356103
CD33	PerCP-Cy5.5	WM53	BioLegend	303414
CD38	PerCP	HB-7	BioLegend	356621
CD69	PE-CF594	FN50	BD Biosciences	562617
HLA-DR	PE-Dazzle594	L243	BioLegend	307654
HLA-DR	SB600	LN3	eBioscience	83-9956-41
HLA-DR	BV421	G46-6	BD Biosciences	562804
FOXP3	AF647	150D	BioLegend	320013
ARG1	APC	A1exF5	eBioscience	17-3697-80
IDO-1	PE	eyedio	eBioscience	12-9477-42
p24	PE	KC51	Beckman Coulter	6604667

Supplemental Table 3. Flow cytometry dyes and antibodies.

Supplemental Table 4

Reagent / Buffer	Manufacturer	Catalog No.
BD Cytofix/Cytoperm™ Kit	BD Biosciences	554714
Tris-EDTA Buffer pH 9	Abcam	ab93684
Citrate Buffer pH 6	Abcam	ab93678
1X Tris-Buffered Saline (TBS)	Fisher Scientific	10776834
Triton X-100	Sigma-Aldrich	9036-19-5
Bovine Serum Albumin (BSA)	Sigma-Aldrich	A9647
Normal Donkey Serum	Jackson ImmunoResearch	017-000-001
Fluoromount G	eBioscience	00-4958-02
OptiView DAB IHC Detection Kit	Ventana	760-700
FOXP3 / Transcription Factor Fixation/Permeabilization	eBioscience	00-5523-00

Supplemental Table 4. Reagents, Buffers and Materials for Flow Cytometry, IF and IHC.

Supplemental Table 5

Marker	Clone / Clonality	Host	Target	Application (Dilution)	Manufacturer	Catalog No.
CD33	SP266 (Monoclonal)	Rabbit	Human CD33	IF (1:100)	Abcam	ab199432
p24	Kal-1 (Monoclonal)	Mouse	HIV p24	IF (1:10)	Dako-Agilent	M0857
CD8	Polyclonal	Rabbit	Human CD8α	IF (1:100)	Abcam	ab4055
GzmB	496B (Monoclonal)	Rat	Human GzmB	IF (1:100)	eBioscience	14-8889-80
HLA-DR	CR3/43 (Monoclonal)	Mouse	Human HLA-DR	IHC (1:500)	Dako-Agilent	M0775
CD33	SP266 (Monoclonal)	Rabbit	Human CD33	IHC (Prediluted)	Cell Marque	760-4952

Supplemental Table 5. Primary Antibodies for Tissue Immunofluorescence (IF) and Immunohistochemistry (IHC).

Supplemental Table 6

Antibody / Reagent	Host	Target	Fluorochrome	Dilution	Manufacturer	Catalog No.
Anti-Rabbit IgG	Donkey	Rabbit IgG	AF488	1:1000	Invitrogen	A-21206
Anti-Rat IgG	Donkey	Rat IgG	AF594	1:500	Jackson ImmunoResearch	712-586-150
Anti-Mouse IgG	Donkey	Mouse IgG	AF647	1:500	Invitrogen	A-31571
DAPI	N/A	DNA / Nuclei	N/A	1:5000	ThermoFisher	D3571

Supplemental Table 6. Secondary Antibodies and Counterstains for IF.

Supplemental Table 7

Target Gene	Oligonucleotide Name	Type	Sequence (5′→3′) / Modifications	Manufacturer
HIV-1 1-LTR	LTR Forward	Primer	5'-TTAAGCCTCAATAAAGCTTGCC-3'	IDT
	LTR Reverse	Primer	5'-GTTCGGGCGCCACTGCTAG-3'	IDT
	LTR Probe	Probe	5'-/56-FAM/CCAGAGTCA/ZEN/ CACAACAGACGGGCA/3IABkFQ/-3'	IDT
Human *CCR5* (Reference)	CCR5 Forward	Primer	5'-GCTGTGTTTGCGTCTCTCCCAGGA-3'	IDT
	CCR5 Reverse	Primer	5'-CTCACAGCCCTGTGCCTCTTCTTC-3'	IDT
	CCR5 Probe	Probe	5'-AGCAGCGGCAGGACCAGCCCCAAG-3'	IDT

Supplemental Table 7. Reagents, Primers and Probes for Cell-associated HIV DNA and RNA Quantification by qPCR.

Supplemental Table 8

Reagent / Kit Name	Manufacturer	Catalog No.
NZY Total RNA Kit	NZYtech	MB13402
SuperScript™ III Reverse Transcriptase	Invitrogen	18080044
Random Primers	Invitrogen	48190011

Supplemental Table 8. Reagents and Kits for RNA Extraction and Reverse Transcription

Supplemental Table 9

Target Gene	Oligonucleotide Name	Type	Sequence (5′→3′) / Modifications	Manufacturer
HIV-1 Ψ *(psi)*	HIV-1 Ψ Forward	Primer	5’-CAGGACTCGGCTTGCTGAAG-3’	Sigma
	HIV-1 Ψ Reverse	Primer	5’-GCACCCATCTCTCTCCTTCTAGC-3’	Sigma
	HIV-1 Ψ Probe	Probe	5'-6-FAM-TTTTGGCGTACTCACCAGT-MGBNFQ-3'	IDT
HIV-1 *env*	HIV-1 env Forward	Primer	5’-AGTGGTGCAGAGAGAAAAAAGAGC-3’	Sigma
	HIV-1 env Reverse	Primer	5’-GTCTGGCCTGTACCGTCAGC-3’	Sigma
	HIV-1 Intact env Probe	Probe	5'-VIC-CCTTGGGTTCTTGGGA-MGBNFQ-3'	IDT
	HIV-1 anti-Hypermutant env Probe	Probe	5’-CCTTAGGTTCTTAGGAGC-MGBNFQ-3’	Thermo Fisher
Human *RPP30* (Amplicon 1)	RPP30_1 Forward	Primer	5’-GATTTGGACCTGCGAGCG-3’	Sigma
	RPP30_1 Reverse	Primer	5’-GCGGCTGTCTCCACAAGT-3’	Sigma
	RPP30_1 Probe	Probe	5'-FAM-CTGACCTGA-ZEN-AGGCTCT-IABkFQ-3'	IDT
Human *RPP30* (Amplicon 2)	RPP30_2 Forward	Primer	5’-CCATTTGCTGCTCCTTGGG-3’	Sigma
	RPP30_2 Reverse	Primer	5’-CATGCAAAGGAGGAAGCCG-3’	Sigma
	RPP30_2 Probe	Probe	5'-HEX-AAGGAGCAA-ZEN-GGTTCTATTGTAG-IABkFQ-3'	IDT

Supplemental Table 9. Primers and Probes for the Intact Proviral DNA Assay (IPDA).
